# Adoptive T‐cell therapies in the clinic

**DOI:** 10.1002/btm2.70086

**Published:** 2025-11-14

**Authors:** Suyog Shaha, Leah Lourenco, Zongmin Zhao, Samir Mitragotri

**Affiliations:** ^1^ John A. Paulson School of Engineering and Applied Sciences Harvard University Allston Massachusetts USA; ^2^ Wyss Institute for Biologically Inspired Engineering Boston Massachusetts USA; ^3^ Department of Pharmaceutical Sciences, College of Pharmacy University of Illinois Chicago Chicago Illinois USA

**Keywords:** adoptive cell transfer, CAR‐T, cell therapy, clinical translation, clinical trials, T cell

## Abstract

T cells, as one of the most abundant immune cell types in the human body, play a central role in therapeutic applications and currently dominate the clinical landscape of cell therapies. Their target specificity and capacity to generate durable therapeutic responses make them a powerful modality for precision therapy. T cell therapies represent a leading frontier in cellular medicine and have been investigated for a broad spectrum of indications, from cancers to autoimmune diseases. Here, we provide a detailed overview of the clinical landscape of T cell therapies. We outline the historical developments that shaped the evolution of T cells into transformative therapies and present a comprehensive analysis of their clinical translation. We discuss key milestones in T cell discovery and provide an overview of the 19 globally approved T cell therapy products. We then examine the core features of these approved products and conduct an in‐depth analysis of 2570 clinical trials involving T cell therapies, identifying three distinct time intervals of growth in clinical activity. Furthermore, we evaluate the evolution of critical trial parameters, such as cell source, disease indication, target selection, and delivery route, highlighting emerging trends and key inflection points. Lastly, we discuss the biological and logistical challenges that limit the broader clinical translation of T cell therapies to new indications and diverse patient populations. Our findings indicate a steady rise in clinical studies and regulatory approvals for T cell therapies, with a notably higher rate of approved products in recent years compared to stem cell therapies. This growth exhibits a phased pattern, with each interval characterized by a major inflection point in scientific advancement and clinical translation. Our discussions will provide a quantitative and contextualized overview of this clinical progress in T cell therapy, offering insights into its current trajectory and future potential as a transformative class of therapeutics.

## Graphical abstract

This review presents a comprehensive analysis of the clinical landscape of adoptive T cell therapies based on 19 approved products and 2570 registered clinical trials involving T cells as therapeutic interventions. We overview the history of clinical development of T cell therapies, highlight the diversity of T cell types, and discuss their wide range of disease indications.

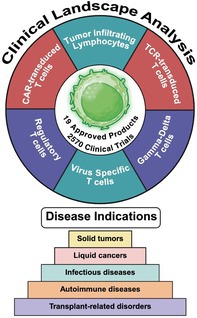

**Translational Impact Statement**

Adoptive T cell therapies have rapidly emerged as a leading frontier in cellular medicine. Their application is expanding through diverse strategies involving multiple T cell types, advanced genetic engineering, and evolving clinical protocols. This review provides a comprehensive overview of the clinical landscape of T cell therapies, highlighting historical milestones, approved products, and key trends in clinical trials over time. We also discuss the scientific and technological challenges shaping the field and offer insights into its current trajectory and future directions.

## 1 INTRODUCTION

Cells are the fundamental units of functional life, and dysfunction at the cellular level is a key driver of many debilitating diseases.^1^ Addressing disease at its root by transferring healthy, corrected, or rejuvenated live cells into the affected patients, thus represents a powerful and increasingly promising therapeutic strategy. In this context, live cell transfer therapies have emerged as a cutting‐edge frontier in modern medicine.^2^ Unlike conventional small‐molecule drugs or biologics, live cells possess the remarkable ability to sense their environment, process complex biological signals, and respond in a programmed, adaptive manner.^3^ These dynamic features allow cell therapies to exert multimodal therapeutic actions that traditional non‐living interventions cannot effectively achieve.^2^, ^4^ Their ability to home to diseased tissues, persist and proliferate in vivo, and interact with the microenvironment through diverse mechanisms has led to profound therapeutic potential.^5^ In several clinical cases, transferred therapeutic cells have remained detectable in patients for over a decade, inducing long‐term, curative effects in cancers with historically poor prognoses.^6^ The practice of live cell therapy, which dates back to the 19th century,^7^ continues to evolve rapidly, fueled by advances in biotechnology and a growing number of clinical investigations. This progress has significantly contributed to the rise of immunotherapy, now a cornerstone of medicine, with numerous agents approved over the past 15 years for a wide range of malignancies.^8^ These therapies have significantly reshaped the standard of care and continue to expand into resistant and refractory diseases, offering new hope to patients with limited options. These advances highlight the growing impact of immunotherapy in biomedicine and the ongoing need for strategies that improve its efficacy, durability and accessibility.

Among the broad array of cell types, stem cells and immune cells are predominantly utilized for cell therapies owing to their small sizes, high counts, and regenerative and immunomodulatory potential.^5^, ^9^ Stem cells, with their capacity for self‐renewal and multilineage differentiation, have a long history of clinical applications, particularly in hematopoietic disorders and regenerative medicine. Our recent review of the clinical landscape of stem cell therapies captures their expanding use across diverse indications.^10^ At the same time, given the immune system's central role in surveillance, defense, and homeostasis, as well as the fact that immune cells account for the majority of the nucleated cell counts in the human body,^11^ it is not surprising that immune cells have become dominant players in the field of cell therapy.^5^ Much of this predominance is driven by the remarkable success of T cell therapies. Since their first description in 1961 by Jacques Miller and colleagues, T cells have been recognized as a centerpiece of the adaptive immune system. They orchestrate antigen‐specific cellular responses and play a critical role in shaping humoral immunity.^12^ Advances over half a century in our understanding of T cell biology, including the identification of distinct effector and regulatory subsets, have laid the foundation for sophisticated T cell engineering platforms. These tools have enabled the development of T cell therapies that target a wide range of diseases, including cancer, autoimmune disorders, graft‐versus‐host diseases, allergies, and infectious diseases. The past decade has witnessed explosive growth in the clinical development of T cell therapies, particularly with the rise of chimeric antigen receptor (CAR) T cell products approved for various hematologic malignancies.^13^ Regulatory approvals have catalyzed substantial investment and innovation in the field, driving the development of next‐generation strategies focused on enhancing manufacturing feasibility, clinical safety, and therapeutic efficacy. The number of T cell‐based clinical trials has expanded rapidly, with some new studies exploring improved treatments for existing indications such as B‐cell‐hematologic malignancies, and others expanding into previously untested disease areas such as autoimmune diseases and T cell‐hematologic malignancies. Additionally, T cell‐based immunotherapy is being actively investigated as a strategy to restore or harness virus‐specific immunity for the prevention and treatment of both infectious and virus‐related non‐infectious diseases.  Notably, clinical exploration in this area began over 20 years ago, highlighting a long‐standing interest in the therapeutic potential of T cell immunity.^14^ This review focuses on capturing these key trends in the rapidly evolving clinical landscape of T cell therapies through the analysis of approved products and clinical trials.

**FIGURE 1:**
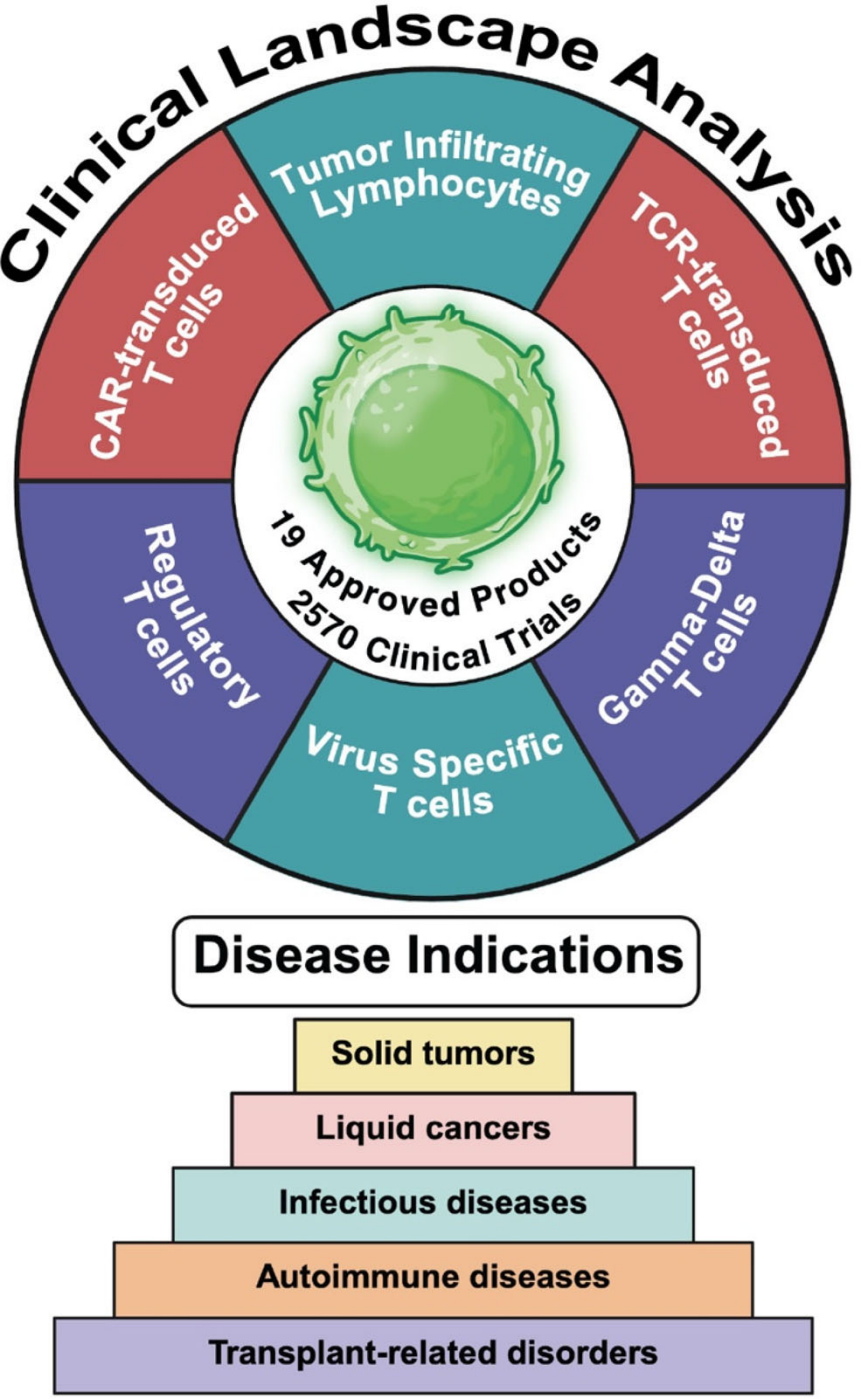
Overview of adoptive T cell therapies in the clinic. This review presents a comprehensive analysis of the clinical landscape of adoptive T cell therapies, based on 19 approved products and 2570 registered clinical trials involving T cells as therapeutic interventions. The T cell types used span a diverse range, from naturally occurring tumor‐infiltrating lymphocytes (TILs) and virus‐specific T cells to regulatory T cells (Tregs), gamma‐delta T cells, and genetically engineered T cells, including TCR‐transduced and CAR‐transduced constructs. The clinical applications of these therapies cover a broad spectrum of diseases, including solid tumors, hematologic malignancies, infectious diseases, autoimmune disorders, and transplant‐related complications.

We begin by highlighting the key milestones over the decades that have shaped the progress of T‐cell therapies. We then analyze 19 distinct approved T cell therapy products identified through a comprehensive search of public databases and regulatory agency records. Next, we critically examine and map 2570 clinical trials investigating the use of T cells as therapeutic modalities, manually curated from an initial pool of over 5000 trials registered in the “ClinicalTrials.gov” database. T cell therapies are now being applied to a broad range of pathologies from cancers, where the endogenous immune system is insufficiently active, to autoimmune diseases, where the immune system is pathologically overactivated (Figure 1). Our analysis reveals three distinct phases in the growth of T cell clinical trial activity, each corresponding to key technological advancements in T cell engineering and clinical application. We structure our analysis around these time intervals: before 2000, 2001–2012, and after 2012, and highlight how the field has evolved and how current investigations align with the scientific and clinical progress of each period. Through this review, we aim to provide a concise historical overview of pivotal events in T cell therapy development, a comprehensive survey of approved products, a temporal analysis of clinical trial trends, and a discussion of key challenges driving current innovation and translational efforts in the field. While an extensive body of literature exists on various aspects of T cell biology, engineering, and clinical application,^15^, ^16^, ^17^ this review offers a focused overview of the clinical landscape of T cell therapies, introducing key concepts where relevant to enhance understanding. It also serves as an update on our previous analyses of the cell and stem cell therapy landscape, this time with an exclusive emphasis on T cell‐based interventions. By incorporating all registered clinical trials involving T cell therapies, we aim to broaden the scope and capture the evolving trajectory of T cell therapies in the clinic.

## 2 BACKGROUND: KEY MILESTONES AND HISTORICAL DEVELOPMENTS

This section outlines key milestones in the development of T cell therapies since their initial discovery in the 1960s. We highlight pivotal historical advances that have shaped the evolution of T cells into powerful therapies.

### 2.1 1960–1980

The early 1960s marked a seminal moment in immunology with the first description of antigen‐specific T cells. Jacques Miller and their colleagues were the first to demonstrate that the thymus plays a critical immunological role, identifying cells originating from this organ.^18^ These cells, later termed T cells, emerged as an essential component of adaptive cellular immunity. Their discovery challenged the prevailing notion of the thymus as a vestigial organ, particularly through observations linking the thymus to susceptibility to virus‐induced leukemia.^19^ The dependency of leukemia induction on the presence or absence of the thymus suggested a key immunological function for this organ. Between 1961 and 1962, further observations of tolerance for foreign skin grafts and the frequent infection‐related deaths in neonatally thymectomized immunodeficient mice reinforced the concept of the thymus as a centerpiece of immune development. These findings led to the identification of thymus‐derived lymphocytes (T cells) as key players in immune surveillance and response.^12^ Subsequent studies established the antigen‐specific role of T cells, showing their cooperation with bone marrow‐derived lymphocytes (B cells) to generate robust antibody responses and their direct involvement in mediating cellular immunity, solidifying their central role in adaptive immunity.^20^ During this time, Southam et al. showed that autologous leukocyte transfer could inhibit the growth of tumor autografts in patients, providing early evidence of the capacity of leukocytes to control cancer cell growth.^21^

As the understanding of T cell biology advanced, clinical observations in the 1970s began to suggest the potential of harnessing the immune cells as a therapeutic modality. Notable cases included the spontaneous regression of advanced gastric cancer in a few patients and an incident where metastatic cancer developed post‐kidney transplantation but disappeared upon cessation of immunosuppressive therapy. These events planted the idea of using a patient's own immune cells, especially T cells, for adoptive therapy.^22^ However, early efforts to study and manipulate antigen‐specific T cell responses in vitro were limited by the short‐lived survival of T cells once removed from the host. To address this, a 1973 study by Symes et al. explored the immunization of pigs with human tissues, followed by the adoptive transfer of sensitized pig lymphocytes.^23^ Although interesting, such approaches were hindered by human leukocyte antigen (HLA) mismatches,^24^, ^25^ which posed significant safety concerns for xenogeneic or allogenic transfer therapies. A vital breakthrough came with the identification of interleukin‐2 (IL‐2) as a T cell growth factor, which enabled the ex vivo expansion and maintenance of T cells. In 1976, Morgan et al. demonstrated that IL‐2‐conditioned media could support the selective growth and long‐term cultures of T cells, maintaining them for over 9 months.^26^ In the late 1970s and early 1980s, significant advances in IL‐2 purification and production made it feasible to carry out extended in vitro experiments with both mouse and human lymphocytes, setting the stage for the development of adoptive T cell therapies.^27^, ^28^, ^29^ Notably, the clinical utility of IL‐2 in modulating lymphocyte responses ultimately led to the FDA approval of high‐dose IL‐2 administration in 1992, marking the first immunotherapy for the treatment of cancer.^30^ One of the earliest demonstrations of the feasibility of ex vivo culturing with IL‐2 for adoptive T cell therapies involved in vitro cultured alloreactive T cells that recognized MHC‐mismatched allogeneic cells. Upon transfer into mice, these T cells retained their alloreactivity and significantly accelerated the rejection of skin grafts, highlighting the potential of ex vivo‐expanded T cells for adoptive therapy applications.^31^

### 2.2 1980–1990

The development of ex vivo culturing protocols for lymphocytes significantly advanced the characterization of T‐cell populations, enabling the identification and isolation of subsets with distinct functional properties and tissue origins. While the cytotoxic nature of T cells was recognized early on following the identification of thymus‐derived T cells,^32^ expanded ex vivo culture studies further reinforced this understanding by demonstrating similar activity in T cells isolated from other sites. Notably, culturing single‐cell suspensions of tumors with IL‐2 for 2 weeks resulted in a predominantly pure lymphocyte population, as non‐lymphoid cells, such as tumor cells, were progressively eliminated through a lymphocyte‐mediated killing.^33^, ^34^ This approach laid the foundation for the isolation of tumor‐infiltrating lymphocytes (TILs), a population enriched for tumor‐reactive T cells.^22^ In 1986, the seminal clinical demonstration of autologous TILs expanded ex vivo and used for adoptive cell transfer in tumor therapy was reported, paving the way for a highly personalized treatment approach.^35^ Around the same time, ex vivo culturing of peripheral blood cells with IL‐2 led to the generation of a predominantly lymphocyte population capable of killing target tumor cells in vitro. These cells, termed lymphokine‐activated killer (LAK) cells, demonstrated anti‐tumor activity in vivo, in mouse models of lung metastases, providing early proof‐of‐concept for their utility in adoptive cell therapy.^35^ While LAK cells, derived from peripheral blood, offered more accessible cell sources, they exhibited limited and less specific cytotoxicity. In contrast, TILs displayed a more activated and proliferative phenotype, with superior specificity, though their efficacy varied.^36^ Nonetheless, the enhanced tumor specificity of TILs made them a more favorable candidate in the early development of personalized adoptive cell therapies.^22^

Although both LAK and TIL cell populations predominantly consisted of T cells, they represented a heterogeneous mixture of lymphocytes.^36^ Advances in the characterization of T cell markers and subsets greatly enriched our understanding of T cell biology, revealing that distinct T cell subsets perform specialized and well‐defined immunological functions.^37^ CD8+ T cells, which primarily recognize antigens presented by MHC‐I, emerged as key effectors with cytotoxic functions, while CD4+ T cells, which interact through MHC‐II, were identified as central to helper function, orchestrating and coordinating broader aspects of the immune response. The helper functions of CD4+ T cells emerged to be diverse depending on the cell lineage. Notably, a lineage of CD4+ T cells has since been recognized as highly diverse, depending on their differentiation into distinct lineages.^38^ As the field advances, a clearer understanding of the heterogeneity and specialized roles of T cell subsets is proving essential to the design of T cell therapies. For example, a key subset first described in 1987^39^ and identified in 1995,^40^ regulatory T cells (Tregs), play a critical role in tolerance for autoimmunity and alloreactivity. Given their immunosuppressive function, Tregs are being actively explored in clinical investigations as a therapeutic strategy to dampen immune responses in the context of autoimmune diseases and organ transplantation,^41^ a shift from earlier efforts that primarily focused on enhancing immune response for tumor elimination.

As the newer T cell subsets were identified, the antigen‐specific nature of T‐cell reactivity sparked significant efforts to understand antigen recognition and receptor structures, representing a pivotal development in advancing T cell‐based clinical applications. In the early 1980s, unique T cell receptor (TCR) complexes were identified on the T cell surface, capable of recognizing and responding to antigens in a highly specific manner.^42^, ^43^, ^44^ Initial mapping of TCRs revealed that most T cells express receptors composed of alpha‐beta chains, now recognized as conventional alpha‐beta T cells. These cells possess a highly diverse TCR repertoire and recognize peptide antigens presented by MHC molecules. Subsequent studies identified another TCR type comprising gamma‐delta chains, forming the basis of the gamma‐delta T cell subset.^45^ Unlike alpha‐beta T cells, gamma‐delta T cells exhibit less TCR diversity and are capable of recognizing certain antigens independently of MHC presentation.^46^ Although the full functional understanding of gamma‐delta T cells is still being elucidated, their distinct immunological properties make them emerging candidates for therapeutic applications,^47^ offering advances in settings where conventional alpha‐beta T cells may be limited. With the key machinery involved in antigen‐specific responses identified and mapped, the importance of costimulatory signals and their associated receptors in driving optimal T cell activity soon became evident. The discovery of co‐receptors and their functional roles helped elucidate how phenotype diversity follows antigen recognition from tolerance induction to potent cytotoxic activity^48^, ^49^ and has been instrumental in guiding T cell engineering strategies to enhance therapeutic efficacy.^50^

### 2.3 1990–2000

In the 1990s, while studies using naturally occurring, ex vivo‐cultured cell populations such as TILs and LAKs were underway, their limited specificity and difficulty in producing therapeutic dosage prompted a shift toward selecting or engineering T cells with defined reactivity against specific targets.^22^ In 1991, the first efforts to identify and clone both shared and mutated tumor‐associated antigens recognized by T cells were reported, marking a pivotal step in targeting melanoma and other tumors.^51^ Shortly after, the first demonstrations of virus‐specific T cells emerged, with evidence for cytomegalovirus (CMV) specificity reported in 1992^52^ and Epstein–Barr virus (EBV) specificity in 1995.^53^ These early discoveries have laid the foundation for the growing body of clinical investigations using donor‐derived virus‐specific T cells, especially for treating hematopoietic stem cell transplant (HSCT)‐related complications and virus‐associated tumors.^54^ Due to the dearth of naturally occurring antigen‐specific T cells, genetic engineering was proposed in the 1990s as a promising strategy to endow T cells with defined specificity, enabling precise recognition and destruction of target cells as antigens were identified.^55^, ^56^ Two years after the initial concept of CARs in a T cell line,^57^ Eshhar and colleagues advanced the field in 1989 by engineering T cells with double‐chain heterodimeric CARs capable of recognizing defined antigens with non‐MHC‐restricted antibody‐like specificity.^58^ This was followed in 1993 by the development of a single‐chain CAR construct, which marked the emergence of the first generation of CAR T cells.^59^ However, the limited clinical efficacy in humans of these early CAR designs in the mid‐1990s led to efforts to optimize their functionality. These efforts culminated in the development of second‐generation CAR T cells in the late 1990s with the incorporation of co‐stimulatory signaling domains,^60^, ^61^ which ultimately paved the way for their clinical success and regulatory approval. Continued improvements in CAR construct design have since been made to improve therapeutic effectiveness and enable the application of CAR T cells across a broader range of disease indications.^13^ Another wave of genetic engineering developed in parallel to CAR technologies, focused on expressing TCRs to enable recognition of defined antigens. Unlike CARs, TCR‐transduced T cells offer the advantage of targeting antigens that are processed and presented on the cell surface, thereby broadening the therapeutic scope. Although several early studies had already explored the safety and feasibility of TCR‐engineered T cells in clinical settings, the first notable clinical use occurred in 2006, when TCR‐transduced T cells specific for the MART‐1 antigen were administered to patients with melanoma to evaluate efficacy.^62^ Since then, the development of affinity‐enhanced TCRs has been implemented to identify suitable TCR constructs to expand the therapeutic scope of this approach.^63^ These advances in genetic engineering during the late 1990s and early 2000s marked a pivotal shift in the T cell therapy paradigm, transitioning from the use of naturally occurring T cells to the emergence of genetically modified T cells as the foundation for therapeutic strategies moving forward.^64^

### 2.4 2000 onwards

The clinical success of genetically engineered T‐cell therapies was not realized until key refinements in clinical protocols were implemented. One such pivotal advancement was the incorporation of lymphodepletion conditioning regimens before the adoptive T cell transfer, a strategy that is now considered an integral component of T cell therapy protocols.^65^ The concept of having a lymphodepleted host to enhance adoptive T cell efficacy dates back to the early 1980s when a study demonstrated that only T cell‐deficient recipients, achieved through thymectomy and irradiation, responded effectively to transferred cells.^66^ However, the mechanistic rationale and clinical significance of this observation were not fully realized until the early 2000s.^66^ A pivotal breakthrough came in 2002 when Dr. Steven Rosenberg's team demonstrated that lymphodepleting chemotherapy prior to T cell infusion enhanced in vivo proliferation, persistence, and tumor infiltration of adoptively transferred T cells.^67^ This study provided critical evidence for the role of the host environment in supporting efficacy and marked a turning point in the clinical development of T cell therapies.^13^ Subsequently, lymphodepletion increasingly became part of adoptive T cell therapy protocols, with conditioning regimens influenced by clinical experience in HSCT.^10^, ^65^ Another exciting advancement in the field has been the optimization of T cell enrichment and expansion, both of which are critical steps in manufacturing consistent and scalable T cell therapy products. One of the impactful innovations is the development of antibody‐coated biomaterials, particularly synthetic beads.^68^ These materials represent a technological breakthrough in the commercialization and standardization of T cell therapies, enabling robust isolation and expansion protocols essential for clinical application.^69^ One of the earliest reports of enriching pure T cell populations using anti‐CD8‐coated beads emerged in 1986.^70^ While efforts to understand the activation of such isolated T cells progressed through the 1990s,^71^, ^72^ a major foundational advancement came in 2003,^73^ with the introduction of a widely adopted protocol for T cell stimulation and expansion using anti‐CD3/CD28‐coated beads. This development marked a significant milestone in T cell manufacturing. Since then, continued efforts have focused on developing improved biomaterials for the isolation, priming, and expansion of T cells to enhance the quality, delivery, scalability, and efficacy of T cell‐based therapies.^74^ These technological and clinical developments of the 2000s led to significant improvements in T cell therapy protocols and set the stage for the clinical success and commercialization of adoptive T cell therapies. The early 2010s marked a pivotal turning point, where years of research and development culminated in transformative clinical outcomes. In 2010, Dr. James Kochenderfer and colleagues reported complete regression in a patient with refractory chronic lymphocytic leukemia following treatment with CD19‐directed CAR T cell therapy.^75^ Shortly thereafter, a similar complete remission was observed in the first pediatric patient with B‐ALL leukemia, a case that had previously carried a grim prognosis.^76^ Both patients had undergone multiple rounds of prior therapies with limited success, yet remained cancer‐free even after a decade following CAR T cell treatment.^13^ These groundbreaking results not only validated the therapeutic potential of engineered T cells but also sparked renewed enthusiasm in the field of adoptive cell therapy. The remarkable efficacy observed in B cell malignancies catalyzed a wave of clinical innovation, transforming the landscape of T cell therapies.^77^ This period marked a critical paradigm shift, from a transitory phase of refinement (2000–2012) to a rapid growth phase, driven by increasing clinical success, key regulatory approvals, expansion into diverse disease indications, and commercial investment in adoptive T cell‐based therapies. In the following sections, we analyze approved products and clinical investigations to map this paradigm shift in T cell therapies, highlighting key trends, milestones, and innovations that have shaped the field.

## 3 APPROVED PRODUCTS

**FIGURE 2:**
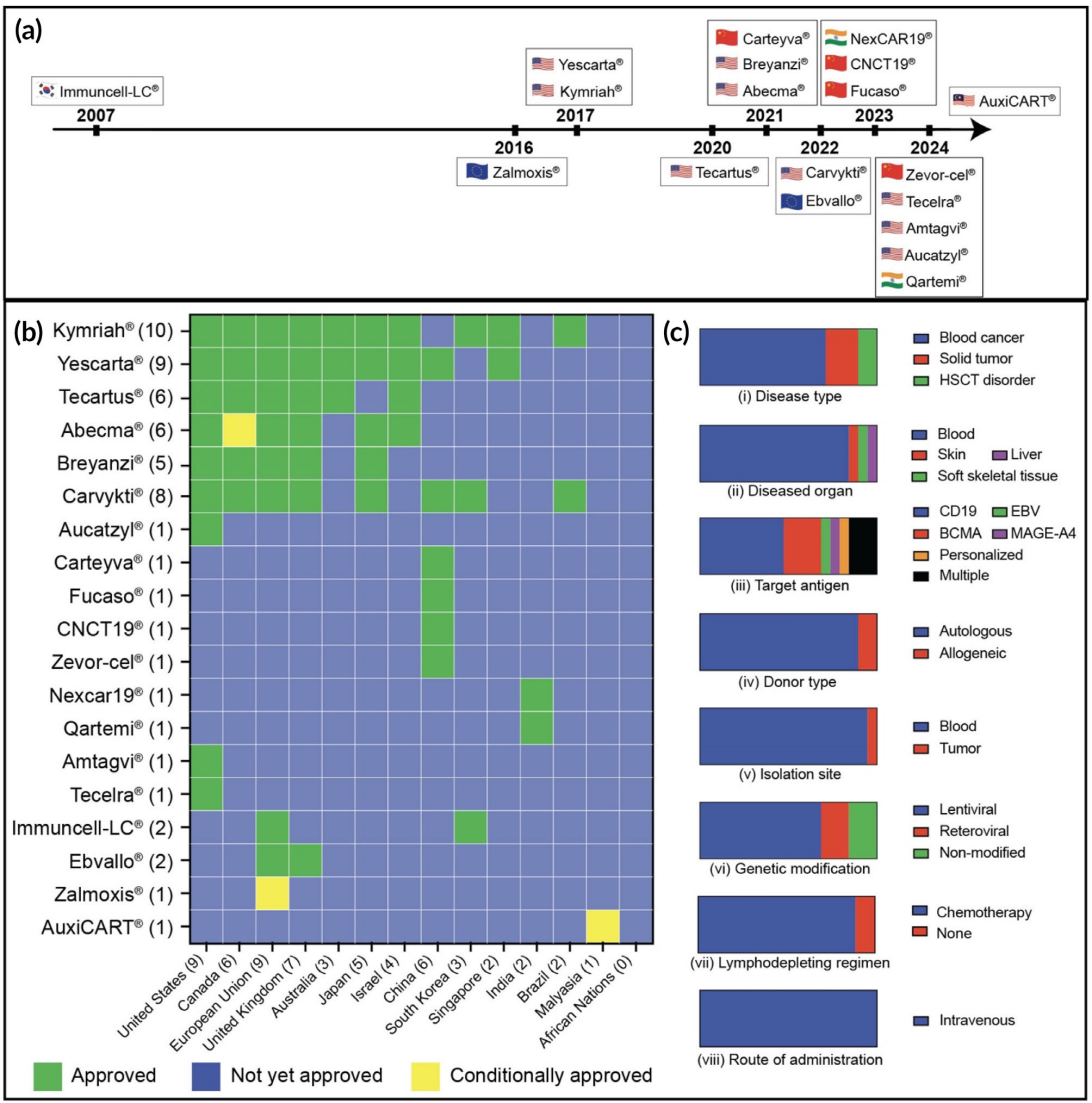
Current landscape of approved T cell therapy products. This review identified 19 distinct T cell therapy products that have received at least one regulatory approval, based on publicly available databases and regulatory agency websites. (a) Timeline showing the year of first regulatory approval for each product. (b) Geographical distribution of regulatory approvals by country or region. (c) Classification of approved products based on 8 key design features.

Rapid progress in the T cell therapy field has been largely driven by the clinical success of T cell therapies over the past decade, as reflected by the growing number of regulatory approvals (Figure 2a). In this section, we present a critical analysis of approved T cell therapy products, identified through a comprehensive search of scientific literature and regulatory agency databases (Table 1). While stem cell therapies currently hold a lead in terms of total approved products (*n* = 27),^10^ T cell therapies (*n* = 19) are rapidly gaining ground and could be expected to surpass stem cell products in the near future. This shift is further evidenced by a temporal transition in the clinical trial landscape, favoring T cell therapies. In 2015, a landscape analysis revealed a dominant presence of stem cell‐related active clinical trials (*n* = 826) compared to adoptive lymphocyte therapies (*n* = 253).^78^ However, by 2021, active T‐cell therapy trials (*n* = 767) had begun to outpace stem cell trials (*n* = 620).^5^ Our most recent analysis further corroborates this trend. While our recent review of stem cell‐related trials identified 800 active trials,^10^ our current analysis, focused on T cell therapies, revealed 1331 active trials, highlighting the substantial growth of clinical research activity of T cell‐based approaches. While some variation may result from time differences in data collection, scope, and method of analysis, the overall trajectory clearly indicates a growing preference for T cell‐based approaches in clinical development. This is further supported by a recent regulatory approval trend: in the past 5 years alone, 14 T cell therapy products have received approval, compared to 8 stem cell therapy approvals during the same period.^10^ Together, these data suggest a paradigm shift in the cell therapy landscape toward adoptive T cell approaches, driven by promising outcomes, increased investment, and expanding indications in oncology and beyond.^79^

**TABLE 1:** Overview of clinically utilized T cell therapy products and associated clinical trials.

Name	Manufacturer/Supplier	T cell type	Target	Gene editing	Delivery Route	Donor type	Associated Clinical trials
Kymriah® (Tisagenlecleucel)	Novartis	CAR‐transduced	CD19	Lentiviral	Intravenous	Autologous	NCT02445248; NCT03568461; NCT03610724; NCT03570892
Yescarta® (Axicabtagene Ciloleucel)	Kite Pharma, Gilead Sciences	CAR‐transduced	CD19	Retroviral	Intravenous	Autologous	NCT02348216; NCT05950802; NCT05108805; NCT04531046; NCT04608487; NCT05800067; NCT05641428
Tecartus® (Brexucabtagene Autoleucel)	Kite Pharma, Gilead Sciences	CAR‐transduced	CD19	Retroviral	Intravenous	Autologous	NCT02348216; NCT05537766; NCT05495464; NCT04880434; NCT03624036
Abecma® (Idecabtagene Vicleucel)	Celgene Corporation, Bristol‐Myers Squibb	CAR‐transduced	BCMA	Lentiviral	Intravenous	Autologous	NCT06045806; NCT05393804; NCT04855136; NCT05032820; NCT04196491; NCT03651128
Breyanzi® (Lisocabtagene Maraleucel)	Juno Therapeutics, Bristol‐Myers Squibb	CAR‐transduced	CD19	Lentiviral	Intravenous	Autologous	NCT03483103; NCT02631044; NCT05873712; NCT05672173; NCT05583149
Carvykti® (Ciltacabtagene Autoleucel)	Janssen Biotech, Johnson & Johnson	CAR‐transduced	BCMA	Lentiviral	Intravenous	Autologous	NCT05201781; NCT06623630; NCT06574126; NCT06550895; NCT05767359
Carteyva® (Relmacabtagene Autoleucel)	JW Therapeutics	CAR‐transduced	C019	Lentiviral	Intravenous	Autologous	NCT05765006; NCT06297408; NCT06365671; NCT06414135; NCT06479356
Fucaso® (Equecabtagene Autoleucel)	IASO Bio	CAR‐transduced	BCMA	Lentiviral	Intravenous	Autologous	NCT05066646; NCT05698303; NCT05201118; NCT05181501; NCT04561557
CNCT19® (Inaticabtagene Autoleucel)	Juventas Cell Therapy	CAR‐transduced	CD19	Lentiviral	Intravenous	Autologous	NCT04011293; NCT04690192; NCT05667506; NCT05930314; NCT06231368
Zevorcel® (Zevorcabtagene Autoleucel)	CARsgen Therapeutics	CAR‐transduced	BCMA	Lentiviral	Intravenous	Autologous	NCT03915184; NCT03975907; NCT06659770; NCT06825845
Nexcar19® (Actalycabtagene Autoleucel)	ImmunoACT	CAR‐transduced	CD19	Lentiviral	Intravenous	Autologous	Jain et.al, Blood, 2024^222^
Qartemi® (Varnimcabtagene Autoleucel)	Immuneel Therapeutics	CAR‐transduced	CD19	Lentiviral	Intravenous	Autologous	Damodar et.al, Blood, 2023^223^
Aucatzyl® (Obecabtagene Autoleucel)	Autolus Therapeutics	CAR‐transduced	CD19	Lentiviral	Intravenous	Autologous	NCT06333483; NCT06173518; NCT04404660
Amtagvi® (Lifileucel)	Lovance Biotherapeutics	Expanded TILs	Multi‐specific	Non‐modified	Intravenous	Autologous	NCT06151847; NCT05727904; NCT05176470; NCT02360579; NCT05640193
Tecelra® (Afamitresgene Autoleucel)	Adaptimmune	TCR‐transduced	MAGE‐A4	Lentiviral	Intravenous	Autologous	NCT05642455; NCT06617572; NCT04044768
ImmunCell‐LC® (Autologous Cytokine Induced Killer Cells)	GC Cell, GC Biopharma Corporation	Cytokine Induced Killer Cells	Multi‐specific	Non‐modified	Intravenous	Autologous	NCT04969731; NCT03220984; NCT00699816; NCT00807027; NCT06620510
Ebvallo® (Tabelecleucel)	Atara Biotherapeutics	Expanded virus specific T cells	EBV	Non‐modified	Intravenous	Allogenic	NCT04554914; NCT03394365; NCT03769467
Zalmoxis® (Nalotimagene Carmaleucel)	MolMed SpA	Suicide gene‐transduced	Multi‐specific	Retroviral	Intravenous	Allogenic	NCT00423124; NCT00914628
AuxiCART®	Auxi Therapeutics	CAR‐transduced	Personalized	Not mentioned	Intravenous	Autologous	‐

### 3.1 First approvals

Since the discovery of T cells in the 1960s, it took several decades of dedicated research and development to achieve the first regulatory approval for a T cell therapy product. Many T cell types have now been approved by regulatory agencies, and the timeline of their initial approvals is outlined in this section (Figure 2a). In 2007, ImmunCell‐LC^80^ became the first approved T cell therapy product, receiving authorization from the Korean FDA. ImmunCell‐LC is an autologous cytokine‐induced killer (CIK) cell therapy, similar to LAK cells, and was approved for use as an adjuvant treatment after tumor resection in hepatocellular carcinoma (HCC). ImmunCell‐LC demonstrated superior recurrence‐free and overall survival in the adjuvant setting for HCC,^81^ and clinical trials (NCT04969731, NCT05053295, NCT00807027) have been initiated to evaluate its efficacy in additional indications. The therapy is manufactured by isolating peripheral blood mononuclear cells from the patients, stimulating them with IL‐2 and aCD3 antibodies, and administering them over up to 16 treatment cycles. In addition to its initial approval, ImmunCell‐LC was also granted approval by the European Medicine Agency (EMA) in 2021 while receiving orphan drug designation for pancreatic cancer, liver cancer, and glioblastoma by the US FDA in 2018, further supporting its therapeutic potential across multiple indications.

In 2016, Zalmoxis^82^ received conditional market authorization from EMA, marking the first genetically modified T cell therapy product to gain regulatory approval for patient access. This therapy involved donor‐derived allogenic T cells genetically engineered to include a suicide gene, allowing their selective elimination if adverse effects occurred. It was administered to patients undergoing HSCT to provide temporary immune protection until full immune reconstitution. However, in 2019, the EMA withdrew conditional approval following unsupportive results from post‐approval phase III clinical trials, which failed to demonstrate a clear benefit in disease‐free survival.^83^ These findings did not provide the confirmatory evidence required to support continued market authorization. This case highlights a critical gap between the initial evidence accepted for conditional approval and the robust clinical validation needed for sustained commercial viability.

The following year, in 2017, the US FDA approved Yescarta and Kymriah, representing the first approvals of genetically modified autologous T cells, specifically CAR T cell therapies, for hematological malignancies. Both products employed the second‐generation CAR constructs targeting CD19, with Yescarta incorporating CD28 and Kymriah incorporating CD137 as their respective costimulatory domains.^84^ These landmark approvals delivered remarkable clinical results^85^, ^86^ and opened the floodgates for global CAR T cell therapy approvals. Subsequent years saw approvals by additional agencies (Canada, UK, Australia, Europe, Japan, Singapore, Israel) and the development of improved CAR T cell therapies. The improved product included Breyanzi, approved in 2021: it introduced a more controlled product by maintaining a 1:1 ratio of CD4+ and CD8+ CAR T cells, intending to improve safety and efficacy^87^; involved determination of dose based on tumor load in the bone marrow and notably did not require a risk evaluation and mitigation strategy (REMS).^88^ In parallel, Abecma became the first CAR T cell therapy product targeting B‐cell maturation antigen (BCMA), expanding the therapeutic landscape beyond CD19. The momentum in the US therapeutic landscape was soon mirrored by developments in China and India, where domestically developed CAR T cell products were successfully approved. Carteyva was approved in China in 2021, followed by NexCAR19 in India in 2023. India, in particular, emerged as a leader by introducing cost‐effective CAR T cell therapies, achieving a 10‐fold reduction in treatment cost,^89^ thereby enhancing accessibility and affordability of CAR T cell therapies in emerging economies and low‐resource settings.

The rapid expansion of CAR T cell therapy approvals has firmly positioned them as a dominant modality within the T cell therapy approval landscape. However, in recent years, other T cell‐based therapies have also gained regulatory recognition, reflecting the growing diversity in therapeutic platforms. In 2022, the EMA approved Ebvallo as the first virus‐specific T cell therapy, marking a key milestone. This therapy involves the selection and expansion of EBV‐specific T cells from HLA‐matched healthy donors, which are then administered as allogeneic T cells to patients post‐HSCT for the treatment of EBV‐associated lymphoproliferative disease.^90^ In 2024, the US FDA approved Amtagvi, marking the first approval of a T cell therapy for solid tumors in the US and the first‐ever regulatory approval of a TIL therapy. This autologous, non‐genetically modified T cell therapy involves the isolation and expansion of tumor‐derived T cells from patients with advanced melanoma, followed by reinfusion.^91^ Later in 2024, another major milestone was achieved with the FDA approval of Tecelra, the first approved TCR‐transduced cell therapy. Tecelra involves genetic modification of autologous T cells derived from peripheral blood, which are modified to express TCR targeting MAGE‐A4, for the treatment of advanced synovial sarcoma.^92^ Altogether, these developments underscore the growing complexity and therapeutic breadth of T cell‐based therapies. Today, the approved landscape includes LAK cells, TILs, virus‐specific T cells, CAR‐transduced T cells, and TCR‐transduced T cells. Meanwhile, gamma‐delta T cells^93^ and regulatory T cells (Treg)^94^ remain promising therapeutic modalities in clinical development but have yet to receive their first regulatory approval. In parallel, CAR T cells, which currently dominate the regulatory‐approved space for hematologic malignancies, have not yet secured approval for solid tumors or autoimmune diseases. However, recent clinical advances in these areas suggest that regulatory approval for these indications may be on the horizon.^95^, ^96^

### 3.2 Approved product analysis

We next examined the specifications of the 19 T cell therapy products that have received at least one regulatory approval to date. This list includes several products that have been granted conditional approvals. We began by analyzing the geographical landscape of these approvals and created a global map to visualize their coverage (Figure 2b). Among the approved products, Kymriah leads with regulatory approvals in approximately 10 regions, followed closely by Yescarta, approved in 9 regions; both are CAR T cell therapies targeting CD19 for B cell malignancies. Carvykti, a BCMA‐directed CAR T cell therapy for multiple myeloma, follows with 8 regional approvals. Notably, the first six CAR T products approved in the United States, all for hematologic malignancies, account for a substantial portion of the global market presence. Other products currently hold approvals in only one or two regions, with ongoing clinical investigations aimed at expanding their regulatory reach and market presence. The US and EU lead globally, each with nine product approvals. However, it is worth noting that Zalmoxis, which received conditional approval in the EU, ultimately failed to secure full regulatory approval. To date, the United Sttates (2 products) and South Korea (1 product) are the only countries with approved T cell therapies for solid tumors. Several countries, including Canada, the United Kingdom, Australia, Japan, Israel, Singapore, South Korea, and Brazil, primarily use products that were originally approved in the United States. In contrast, China^97^ and India^98^ are emerging as development hubs in the T cell therapy space, with four and two domestically developed products, respectively. Malaysia has also entered the landscape with AuxiCART, a homegrown therapy that has received conditional approval for case‐by‐case administration. Unfortunately, there are currently no approved T cell therapy products in any African nations, likely due to a combination of resource‐limited healthcare infrastructure and a higher allocation of medical resources to address the treatable infectious disease burden.^99^ As global access to advanced therapies expands, efforts to bridge these gaps will be critical. Overall, the global landscape is currently dominated by CAR T cell therapies targeting hematologic cancers, with expanding reach across multiple regulatory regions.

We conducted a detailed analysis of the workflow associated with each of the 19 approved T‐cell therapy products and identified key patterns across critical factors governing their manufacturing and application (Figure 2c). Blood cancers remain the dominant indication, accounting for 71% of approved products, followed by solid tumors (18%) and HSCT‐related complications (11%). In terms of disease pathology, 84% of approved therapies target hematological pathologies, while skin, liver, and soft skeletal indications each represent approximately 5.2% of the total. This distribution highlights the current success of T cell therapies in blood‐related disorders, with limited but emerging progress in solid tumor indications. Antigen specificity remains a central determinant of T‐cell therapy design and efficacy. CD19, a B cell marker, is the most frequent target antigen, featured in 47% of approved products. BCMA, expressed on plasma cells, is the next most common target, appearing in 21% of products. Notably, 16% of approved therapies are designed to target multiple antigens without a single dominant target, while 5.2% are personalized and directed against patient‐specific antigens. A majority, 89% of the products, utilize autologous T cells, largely due to the MHC restriction inherent to T cell reactivity, which poses a significant risk of graft‐versus‐host disease (GvHD) in mismatched allogeneic settings.^100^ The remaining 11% of products use allogenic T cells, primarily to treat HSCT‐related complications during the period when patients are awaiting immune reconstitution. In such cases, the immunocompromised state reduces the risk of GvHD, making allogeneic T cell therapies feasible for short‐term therapeutic action. Peripheral blood is the predominant source for T cell isolation, with 95% of approved products utilizing it as a primary source. Only one product (5%) sources T cells from tumor tissue, reflecting the rarity of alternative tissue‐derived T cell therapies. Although antigen‐specific T cells are typically present at low frequencies in peripheral blood,^101^ the emergence of genetic engineering has enabled their redirection and use to recognize and target specific antigens effectively. In addition to such development, peripheral blood offers significant logistical advantages, making it the most clinically feasible source for T cell collection. The ease of collection with sophisticated ex vivo modification technologies has solidified peripheral blood as the standard source for therapeutic T cell manufacturing.

While 16% of approved T cell therapy products utilize genetically unmodified T cells, the majority, 84%, are genetically modified, incorporating modifications such as CARs, TCRs, or suicide genes to enhance specificity or safety. Among the genetically modified products, 81% use lentiviral vectors for gene delivery, while the remaining 19% rely on retroviral vectors. Lentiviral vectors are preferred for engineering due to their ability to transduce both dividing and non‐dividing cells, making them more efficient in modifying T cell populations.^102^ In contrast, retroviral vectors only transduce in a dividing state, limiting their utility.^103^ Moreover, lentiviral vectors are associated with a lower risk of insertional mutagenesis, a safety concern that has gained attention in the context of T cell therapies. This concern is particularly relevant given the rare but possible risk of insertional events leading to oncogenic transformation, such as the development of secondary primary cancers, like T cell lymphoma.^104^ However, it is important to note that the link between T cell therapies and such secondary cancer risks remains unclear and under active investigation. These advantages have positioned lentiviral vectors as the dominant platform for clinical‐grade T cell engineering. As discussed in the context of historical developments, a lymphodepletion preparative regimen has become an invaluable component of T cell therapies, as it facilitates engraftment and persistence of transferred T cells by creating space in the immune compartment and mildly reducing tumor burden before T cell therapy.^105^ Currently, 90% of approved T cell therapy products require patients to undergo lymphodepleting chemotherapy prior to the adoptive cell transfer. This regimen typically consists of a combination of cyclophosphamide and fludarabine, with dosing strategies largely informed by extensive clinical experience in HSCT.^10^ Although lymphodepleting chemotherapy can be debilitating for patients, it significantly enhances T cell expansion, persistence, and overall therapeutic efficacy following infusion.^106^ Thus, it remains a core component of most approved T cell therapy protocols despite its associated toxicities. Unsurprisingly, all approved T cell therapy products are administered intravenously, which aligns with both the predominance of blood‐related malignancies as target indications and the antigen‐specific activity of T cells. The intravenous route offers the advantage of efficient systemic distribution, ease of administration, and compatibility with existing clinical workflows for other therapeutic modalities. However, the risk of systemic toxicities, such as cytokine release syndrome and immune effector cell‐associated neurotoxicity, remains a central delivery challenge in patient management. These limitations have prompted interest in locoregional delivery strategies,^107^ particularly for solid tumors anatomically confined or immune‐privileged sites, such as the brain, where localized delivery may greatly improve efficacy while minimizing systemic toxicity.

The real‐world implementation of approved T cell therapy products continues to generate valuable insights into their long‐term safety and efficacy. The US‐FDA has approved all CAR T cell therapies with the requirement of 15 years of long‐term follow‐up to monitor for delayed adverse events, including the potential development of secondary primary malignancies following the administration of genetically engineered cells. In cases where new malignancies are observed, this requirement may extend to lifelong monitoring, underscoring the importance of sustained post‐marketing surveillance in evaluating the full risk profile of these advanced therapies.^108^ An analysis of non‐relapse‐related deaths following CAR T cell therapy revealed that infections accounted for the highest proportion of cases (51%), followed by secondary malignancies, which represented the second most common cause (7.8%).^109^ Reports of CAR‐positive T cell lymphomas emerging as secondary malignancies in patients treated with CAR T cell products have raised important safety concerns.^110^ Although such cases remain extremely rare, with the US‐FDA reporting 33 cases among over 30,000 treated patients, they have prompted regulatory action.^104^ In response, the FDA has mandated the inclusion of a black box warning on the package inserts of all approved CAR T cell therapies, explicitly stating the risk of therapy‐related secondary cancers. Follow‐up data analyses indicate that the risk of secondary cancers associated with CAR T cell therapy remains low, especially when compared to other cancer treatments.^108^ However, these rare cases of T cell malignancy have prompted more thorough investigations to determine whether a causal link exists between CAR T cell therapy and the development of such malignancies.^111^ The balance between achieving persistent engineered T cells for durable therapeutic responses and mitigating the long‐term risk of unexpected adverse effects, including oncogenic events, remains a key limiting factor in the development of T cell therapies.^112^ Despite this, the overall clinical benefits of these approved therapies remain substantial and clearly outweigh the associated risks for their intended approved use.

Altogether, our analysis of approved T cell therapy products highlights the key factors that have contributed to their clinical success and offers insights into the design principles shaping ongoing clinical development. However, it does not capture the unsuccessful or less effective approaches that were previously explored before reaching regulatory approval. To address this gap in our understanding, we next turn to the clinical trial landscape to examine investigational strategies temporally and further decipher the evolving trajectory of T cell therapies in the clinic.

## 4 CLINICAL TRIAL ANALYSIS

We conducted a systematic search of the “ClinicalTrials.gov” database to identify clinical trials involving adoptive T cell therapies. Using the keyword “T cell”, we filtered for interventional studies with the recruitment status marked as active for “recruiting/active, no recruiting/not yet recruiting/enrolling by invitation”, completed for “completed”, adjourned for “terminated/suspended,” and unknown for “unknown.” This initial search yielded over 5000 trials. Following manual review and careful screening, we identified 2570 trials in which adoptive T cell transfer was included as a component of the therapeutic intervention. While this database may not capture every existing trial, it offers a reliable and comprehensive resource for identifying representative trends in the field. During our manual analysis, we recorded key parameters of each selected trial, including start year, trial status, phase, sponsor, disease indication, pathological site, delivery route, T cell type, donor type, tissue source, ex vivo modification technique, target specificity, and preparative conditioning—factors critical to the clinical trial analysis of adoptive T cell therapies. Using these defining characteristics, we conducted a detailed analysis of the clinical trial landscape to map the temporal evolution and development of T cell therapies in this setting.

### 4.1 Start year

**FIGURE 3:**
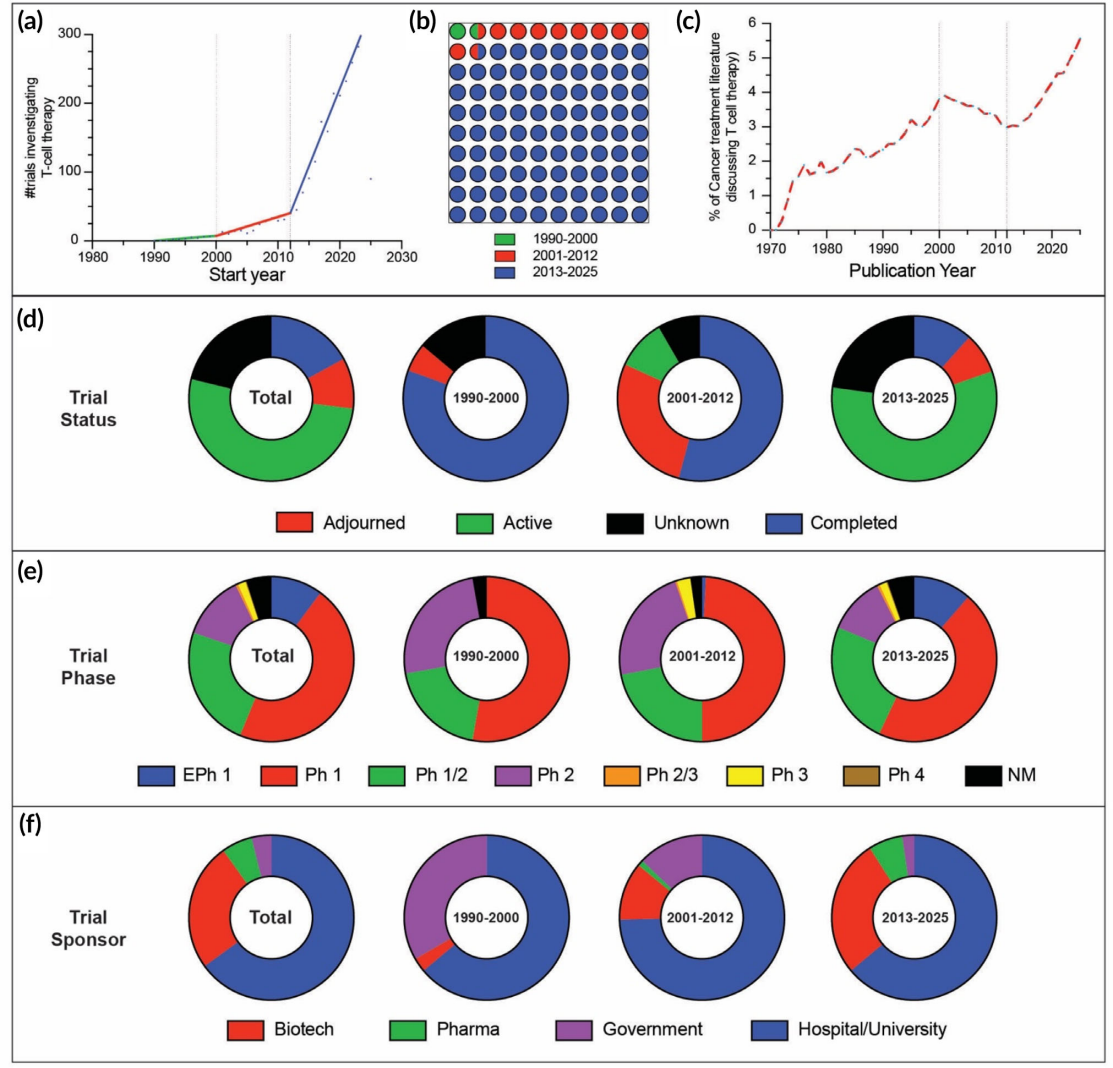
The landscape of T cell therapy clinical trials: Trial specifications. This review analyzed 2570 clinical trials involving T cells as a therapeutic intervention, identified from the ClinicalTrials.gov database. (a) Annual distribution of trial initiations, highlighting three distinct phases of growth based on differing slopes: 1990–2000 (green), 2001–2012 (red), and 2013–2025 (blue). Vertical lines in 2000 and 2012 denoting inflection points aligned with T cell therapy clinical milestones, (b) Distribution of trials across three major intervals based on the growth phase. (c) Percentage of cancer treatment literature referencing T cell therapies, plotted by year of publication, with vertical lines in 2000 and 2012 denoting inflection points aligned with clinical trial trends. (d)–(f) Distribution of trials by key specifications across all stages and individual intervals: (d) Trial phase, (e) Trial status, (f) Trial sponsor type. NM, not mentioned.

Driven by the development of approved T cell therapies, the clinical trial landscape for T cell therapies has expanded rapidly over the past decade. This growth became even more evident when we plotted the number of trials initiated each year (Figure 3a). Our analysis revealed three distinct time intervals (1990–2000, 2001–2012, and 2012–2025) characterized by markedly different growth rates. Notably, the years 2000 and 2012 emerged as key inflection points, each marked by sharp increases in the number of initiated trials and reflecting a significant paradigm shift in the development and implementation of T cell therapies. Around 2000, the field witnessed the emergence of second‐generation CAR T cells, which demonstrated enhanced efficacy and introduced genetic engineering as a viable clinical strategy for T cell modification. Concurrently, the incorporation of lymphodepleting regimens into clinical trial protocols helped improve engraftment and persistence, marking the beginning of a transitional phase where these new strategies were being explored alongside traditional T cell approaches. In contrast, the period around 2012 was defined by the clinical success of the second‐generation CAR T cells in patients with B‐cell malignancies that had otherwise poor prognoses. These early yet compelling results elevated CAR T cell therapy from a preclinical concept to a cornerstone of the clinical landscape of T cell‐based therapies. The period from 1990 to 2000 accounts for only 1.4% of all identified trials, while the 2001–2012 interval represents 10.3%. In contrast, the 2013–2025 period encompasses a striking 88.3% of all trials, highlighting the logarithmic growth in clinical activity across these time frames (Figure 3b). To understand how this clinical momentum has been reflected in the scientific literature, we examined publications related to cancer treatment, where T cell therapies have been predominantly explored, accounting for approximately 85% of clinical trials and 89% of approved products in this space. Specifically, we analyzed the proportion in PubMed publications associated with the keywords “Tumor treatment AND T cell therapy” relative to all publications tagged with “Tumor treatment,” plotted by year of publication (Figure 3c). Interestingly, this analysis revealed a similar three‐phase pattern but with some nuanced trends. Till 2000, there was a steady increase in the percentage of T cell‐related publications. However, this upward trend reversed after 2000, marking a relative decline in focus on T cell therapies in the literature. This shift may be attributed to the emergence and clinical success of monoclonal antibody‐based immunotherapies such as Rituximab, Trastuzumab, Ipilimumab, and Pembrolizumab, which gained significant momentum during this period.^113^ As these therapies rapidly advanced in the clinic and demonstrated substantial therapeutic efficacy, the spotlight in cancer immunotherapy was temporarily reduced for T cell therapies, which were still in the early stages of establishing themselves as a viable therapeutic strategy. Notably, around 2012, this trend began to reverse again, leading to a renewed and accelerated surge in T cell therapy‐focused cancer treatment literature, surpassing the growth observed before 2000 and paralleling the sharp rise in the clinical trial activity of T cell therapies. Given these stark differences across the three time intervals, we further analyzed key defining factors by segmenting the trial accordingly and examining each period in greater detail.

### 4.2 Trial status and phase

Altogether, 51.8% of the trials have active recruitment status, reflecting a strong level of ongoing activity and investment in T cell therapy research. Additionally, 17% of trials have been completed and 10% have been adjourned, providing a valuable body of clinical experience that continues to inform and share the development of T cell therapies (Figure 3d). Meanwhile, 21.2% of trials are listed with an unknown status. As expected, clinical trial statuses vary significantly across the three time intervals, offering a useful framework for analyzing key trends. In the early period (1990–2000), 81% of trials were completed, with no active trials ongoing. In the transitory period (2001–2012), 54% of trials were completed, while a notable 28% were adjourned. In contrast, the most recent period (2013–2025) is marked by 58% active trials and only 12% completed. Notably, the 28% adjournment rate during the transitory period stands out, especially when compared to 6% and 8% in the early and late intervals, respectively. This could suggest that the 2001–2012 period involved substantial trial‐and‐error learning as the field grappled with the technical, financial, and logistical complexities of translating T cell therapies into the clinic. These challenges likely contributed to higher attrition rates and provided essential insights that paved the way for the more refined strategies seen in recent years.

The clinical phase distribution of T cell therapy trials reflects the typical trajectory of drug development, where only a limited number of strategies progress from early‐stage safety and feasibility studies to late‐stage efficacy trials (Figure 3e). Notably, 80.3% of all T cell therapy trials are concentrated in the early stages (Early Phase 1, Phase 1, Phase 1/2) while only 2.2% have reached late‐stage clinical development (Phase 3 and Phase 4). In the early period (1990–200), the majority of T cell therapy trials were concentrated in the early stages (72.2%), with no trials initiated in late‐stage development (Phase 3 or 4), likely reflecting the lack of promising outcomes from early T cell strategies during this period. This began to shift during the transitory period (2001–2012), where 3.1% of trials reached late‐stage phases, although no Phase 4 trials were recorded. In the late period (2013–2025), 2.1% of trials were late‐stage development, while a notable increase in Early Phase 1 trials (also referred to as Phase 0) emerged, accounting for 11.4% of all trials. These early phase 1 trials are exploratory, designed to assess the feasibility of clinical manufacturing and clinical workflow, a critical consideration for resource‐intensive T cell therapies. The increased emphasis on this exploratory phase is likely to contribute to reduced trial adjournment rates seen in the transitory period by enabling better refinement in workflow and regulatory readiness, ultimately supporting more robust investigational new drug (IND) applications for Phase 1 trial initiation.^114^

### 4.3 Trial sponsor

A sponsor of a clinical study is responsible for its initiation, management, and funding. To understand the drivers of T cell therapy development, we analyzed the distribution of clinical trial sponsors. Sponsors were categorized into four groups: industry‐based (biotechnology or pharmaceutical companies) and academic (government agencies or hospital/university institutions). This categorization allowed us to identify the key sectors and regions propelling progress in the clinical landscape of T cell therapies. Overall, academic institutions, including government agencies and hospitals/universities, dominate the sponsorship profile, accounting for 68.9% of all trials. In contrast, industry‐sponsored trials made up 31%, with biotech companies contributing 25% and pharmaceutical companies 6% (Figure 3f). These findings highlight the pivotal role academic sponsors have played in transforming T cell therapies from concept to clinical reality. This trend becomes even more apparent when examining the temporal evolution of sponsorship profiles. In the early period (1990–2000), academic sponsors were responsible for 97.3% of trials, while biotech accounted for only 2.7% and pharmaceutical companies had no presence. This underscores the critical importance of funding academic research as the foundation for early innovation, which subsequently attracts industry interest for further development and commercialization. In the transitory period (2001–2012), academic dominance persisted, sponsoring 87.5% of trials, though industry involvement began to increase. The latest period (2013–2025), which marked a boom in T cell therapy activity, witnessed a 2.6‐fold increase in industry‐sponsored trials, reaching 33.8%. This shift reflects the growing engagement of biotech and pharma companies in T cell therapy development, as clinical success and regulatory milestones have de‐risked investment in this space. In summary, while academia continues to lead in clinical investigation and innovation, the industry is rapidly catching up, playing an increasingly influential role in advancing T cell therapies. The evolution of T cell therapy is a powerful testament to the importance of sustained investment in academic research as a catalyst for next‐generation medical breakthroughs.

### 4.4 T cell type

**FIGURE 4:**
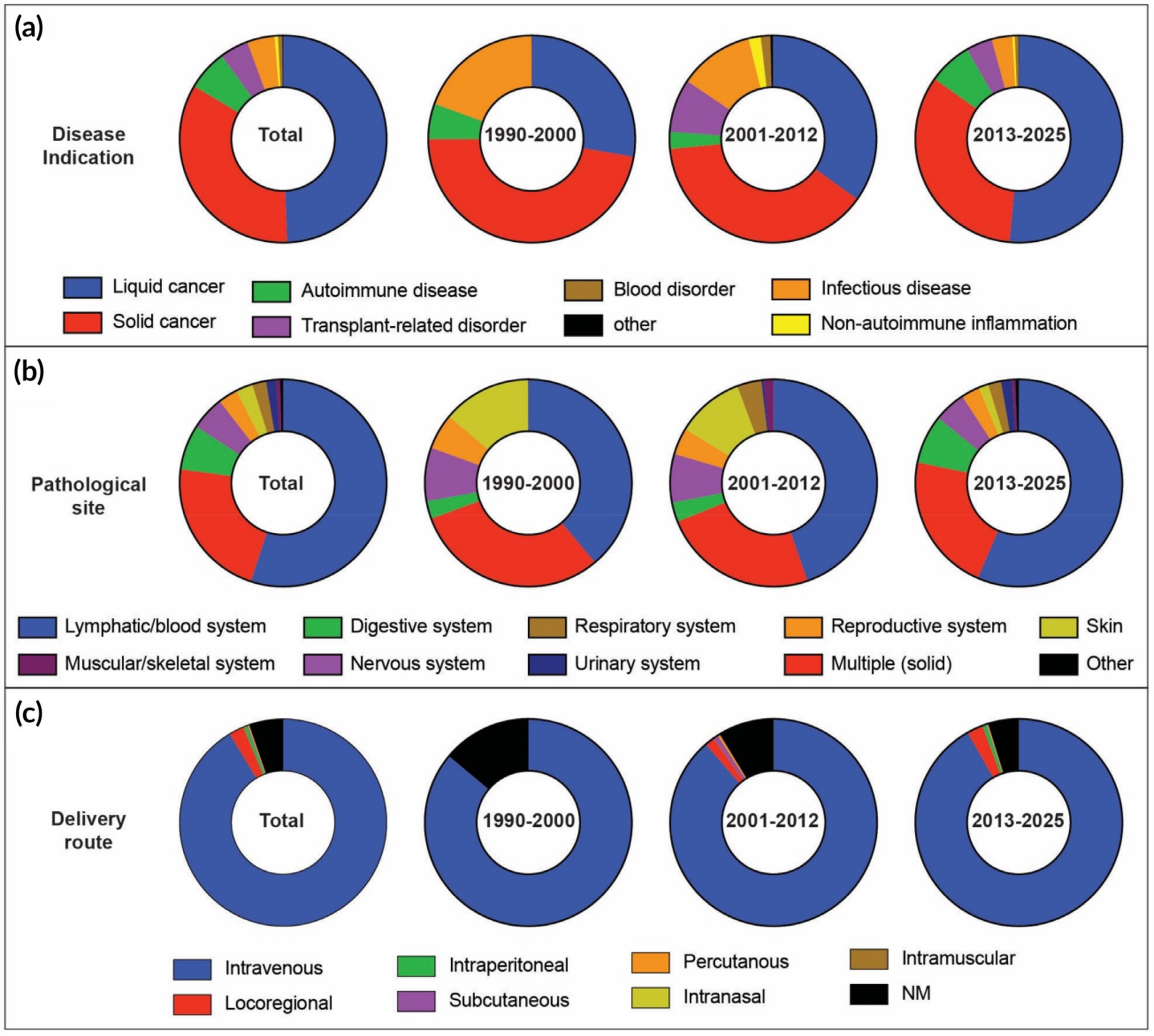
The landscape of T cell therapy clinical trials: Target site. (a)–(c) Distribution of trial specification dictated by target site across all stages and individual intervals: (a) Disease indication, (b) pathological site, (c) delivery Route. NM, not mentioned.

The technological evolution of the T cell therapy landscape is most evident when examining the distribution of T cell types explored across different time intervals (Figure 4a). The dominance of CAR T therapy is well recognized, accounting for 73.2% of all approved T cell therapy products. Reflecting this, our analysis found that 64% of clinical trials have utilized CAR T cells as the intervention, making them the most extensively studied T cell type. This is followed by T cells that are solely primed and expanded, used in 17.9% of trials, which also represent 10.5% of approved products. In contrast, TCR‐transduced T cells account for only 7% of trials, while virus‐specific T cells appear in just 4.9% of trials. This distribution stands in stark contrast to earlier phases of T cell development. In the early stage, 77.7% of trials involved solely primed‐expanded T cells, including strategies based on TILs and LAK cells. Additionally, 16.6% of trials focused on virus‐specific T cells. Researchers primarily relied on naturally occurring T cells for adoptive cell therapy formulations, reflecting the fact that genetic engineering technologies for T cells were still in their infancy following their initial emergence in the late 1990s.^115^ Notably, no CAR T cell trials were initiated during this period, and only 2.7% of trials involved TCR‐transduced T cells. Pilot studies conducted at the NCI in the late 1990s demonstrated the safety and feasibility of the adoptive transfer of unedited primed‐expanded T cells.^116^ However, the effectiveness was limited due to the inability to generate sufficient numbers of effector T cells with specificity for targeted antigens necessary for robust therapeutic activity. This challenge catalyzed the development of rapid expansion protocols (REPs), which became a major focus of the field during this period to accelerate clinical translation. During this period, the REP process evolved significantly from IL2‐supplemented media to the use of soluble or plate‐bound anti‐CD3 monoclonal antibodies, allogeneic feeder cells expressing co‐stimulatory molecules, and eventually, antibody‐coated beads.^117^ The integration of these components into clinical protocols became widely adopted as a strategy to enhance T cell yield. Importantly, this era also saw a marked reduction in the ex vivo culture time, allowing for more efficient clinical manufacturing and faster treatment times.

The transitory period (2001–2012) marked the emergence of genetically engineered T cells in the clinical landscape, indicated by a modest increase in the proportion of trials utilizing CAR T cells (11.7%) and TCR‐transduced T cells (6.8%). Despite these new developments, unedited primed‐expanded T cells continued to dominate, accounting for 59% of trials, followed by virus‐specific T cells, which comprised 15.2% of trials. Only a few strategies using unmodified T cells advanced to Phase 3, including ImmunCell‐LC for brain tumors (NCT00807027) and liver cancer (NCT00699816), CMV‐specific T cells for post‐HSCT CMV infections (NCT01077908), and myelin‐reactive T cells for multiple sclerosis (NCT00228228). However, except for ImmunCell‐LC in liver cancer, none of the other approaches demonstrated significant clinical promise,^81^, ^118^, ^119^ contributing to the drop in interest in such approaches in the upcoming period. Meanwhile, several TCR‐transduced T‐cell therapies advanced to Phase 2 clinical trials (NCT00910650, NCT01352286, NCT00509288) and showed preliminary signs of efficacy in solid tumors, keeping interest in the platform alive.^120^ The true inflection point, however, came with CAR T cell therapies targeting the CD19 antigen, which, though still in Phase 1/2 during this period (NCT01626495, NCT00924326, NCT00466531), demonstrated remarkable preliminary efficacy that would soon redefine the field of adoptive T cell therapy. This breakthrough was made possible through the incorporation of lymphodepleting regimens and the use of second‐generation CAR constructs, which significantly enhanced in vivo expansion, persistence, and effector activity.^121^ This transformative development during the transitory period led to a complete reshaping of the clinical landscape of T cell therapies. The share of CAR T cells rose dramatically from 11.7% in 2001–2012 to 71.2% in the 2013–2025 period. Initially, the majority of CAR T cell therapies in this later phase targeted B cell‐related hematologic malignancies, particularly B cell leukemias and lymphomas. However, as clinical success mounted and multiple products received regulatory approval, the therapeutic rationale for CAR T cells began to expand beyond oncology.^95^ In recent years, especially over the past five, CAR T cells have been investigated in non‐malignant B‐cell‐related disorders such as autoimmune and inflammatory diseases. A notable example came in 2022, when a pilot clinical study showed a resolution of systemic lupus erythematosus (SLE) symptoms and reversal of organ damage markers in a patient treated with CAR T cell therapies.^122^ With multiple clinical studies reporting similar outcomes, this represents a promising new frontier for CAR T cell therapy in the upcoming period.^13^, ^123^ Simultaneously, solid tumors have emerged as a key area of interest for CAR T cell therapy, though clinical outcomes have been mixed and largely disappointing compared to liquid cancers.^96^ While significant efforts are underway to overcome the barriers that solid tumors pose to CAR T cell efficacy, TCR‐transduced T cells, predominantly targeting solid tumors,^5^, ^96^ have maintained a consistent presence. They still account for 7% of clinical trials, similar to their share during the transitory period. Additionally, gamma‐delta T cells, which operate via an alternative mechanism of action compared to traditional alpha‐beta counterparts, have show early promise in the treatment of solid tumors, particularly for off‐the shelf product development with allogenic use feasibility. They hold a 2.1% share of trials, most of which are recently started. Additionally, gamma‐delta T cells, which operate via an alternative mechanism of action compared to traditional alpha‐beta counterparts, have shown early promise in the treatment of solid tumors, particularly for off‐the‐shelf product development with allogenic use feasibility.^124^ They currently account for 2.1% of clinical trials, the majority of which have been initiated in recent years. Beyond this development, T‐cell therapies continue to be explored in transplant‐related complications such as graft alloreactivity, GvHD, and opportunistic infections, all of which remain critical clinical challenges.^41^, ^94^ In this space, regulatory T cells and virus‐specific T cells are the primary modalities under investigation. Notably, Tregs are also being actively explored for the treatment of autoimmune and inflammatory diseases, with efforts to enhance their suppressive function through CAR transduction gaining momentum.^94^, ^120^, ^125^ Meanwhile, virus‐specific T cells are being employed not only for virus‐associated cancers^54^ but also as platforms for genetic engineering to target tumor antigens, owing to their enhanced persistence.^126^, ^127^ Interestingly, the proportion of Treg‐based trials has remained stable at ~3% between the 2001–2012 and 2013–2025 intervals. In contrast, the share of virus‐specific T cells has declined significantly, from 15% in the transitory period to just 3.6% in the most recent period, reflecting a shift in clinical focus toward genetically modified T cell therapies and other emerging opportunities. The choice of T cell type forms the core of the therapeutic strategy and influences trends across all other aspects of therapy development.

### 4.5 Source

T cells play a central role in eliciting GvHD, making avoidance of HLA‐mismatching a key consideration in T cell therapy design.^128^ As a result, autologous cell sources, where the patient's own cells are used, are preferred in the majority of T‐cell therapy trials, accounting for 60.7% of all trials. This prevalence of autologous cell sourcing has remained consistent across periods, with 55.6% of trials from 1990 to 2000, 55.9% from 2001 to 2012, and 61.8% of trials from 2012 to 2025 using autologous cells (Figure 4b). However, cell source preference is heavily influenced by T cell type. Among the trials using unedited expanded‐primed T cells, 54.7% used autologous sources. This preference is more pronounced in genetically engineered T cell therapies, with 65.8% of CAR T cell trials and 70.8% of TCR‐transduced T cell trials utilizing autologous sources. In contrast, only 43.3% of trials using Tregs, 24.4% of trials using gamma‐delta T cells, and 24.6% using virus‐specific T cells relied on autologous cell sources, reflecting the greater feasibility of allogeneic off‐the‐shelf approaches in these modalities. Given these distinctions, recent years have seen a growing interest in developing universal, allogenic T‐cell products. Notably, of all trials using allogenic cell sources, a substantial portion has been initiated in the last 5 years: 70% of TCR‐transduced T cell trials, 80.6% of gamma‐delta T trials, and 78.9% of CAR T cell trials with allogenic sourcing began during this period. While all currently approved CAR T cell therapies are autologous, early studies using allogeneic CAR T cells have shown encouraging potential (e.g., NCT02808442 and NCT02746952). In a Phase 2 clinical study of Kymriah (NCT02435849), it was reported that 18.5% of patients were unable to receive therapy due to challenges with autologous cell manufacturing.^86^ These limitations have sparked significant enthusiasm for the development of universal, off‐the‐shelf CAR T cell products, which could provide timely treatment options for patients who are otherwise unable to undergo autologous approaches.^129^ This trend underscores a broader shift toward scalable, off‐the‐shelf solutions in T cell therapy.

Although a significant portion of trials (43.8%) do not specify the tissue source for T cell harvesting, peripheral blood is the most commonly reported source, mentioned in 54% of trials overall, representing 96% of all trials with a clearly identified source. Peripheral blood is a practical and abundant source of T cells, making it the preferred option for T cell isolation.^130^ Its use has remained remarkably consistent across time, with 55.5% of trials from 1990 to 2000, 55.7% from 2001 to 2012, and 53.8% of trials from 2013 to 2025, utilizing peripheral blood as the isolation site for T cells (Figure 4c). The exponential rise of CAR T cell therapies has not significantly altered this trend, as these therapies also commonly utilize peripheral blood as the source of T cells. Interestingly, the use of tumor tissue as a T cell source, more common in earlier strategies such as TIL‐based therapies, has declined slightly over time. It was reported in 2.8% of trials during 1990–2000, dropping to 1.5% in 2001–2012, and remaining at 1.5% in 2013–2025. Notably, a few recent trials (NCT05908643, NCT06716619, and NCT06302062) have been initiated to explore tumor‐draining lymph nodes as a source of T cells, based on the hypothesis that the lymph nodes contain a higher proportion of tumor‐specific T cells than those within the tumor microenvironment and these cells often exhibit a less exhausted phenotype.^131^ Altogether, these trends indicate a broader inclination toward utilizing more accessible and scalable T cell sources in clinical practice.

### 4.6 Disease indications

**FIGURE 5:**
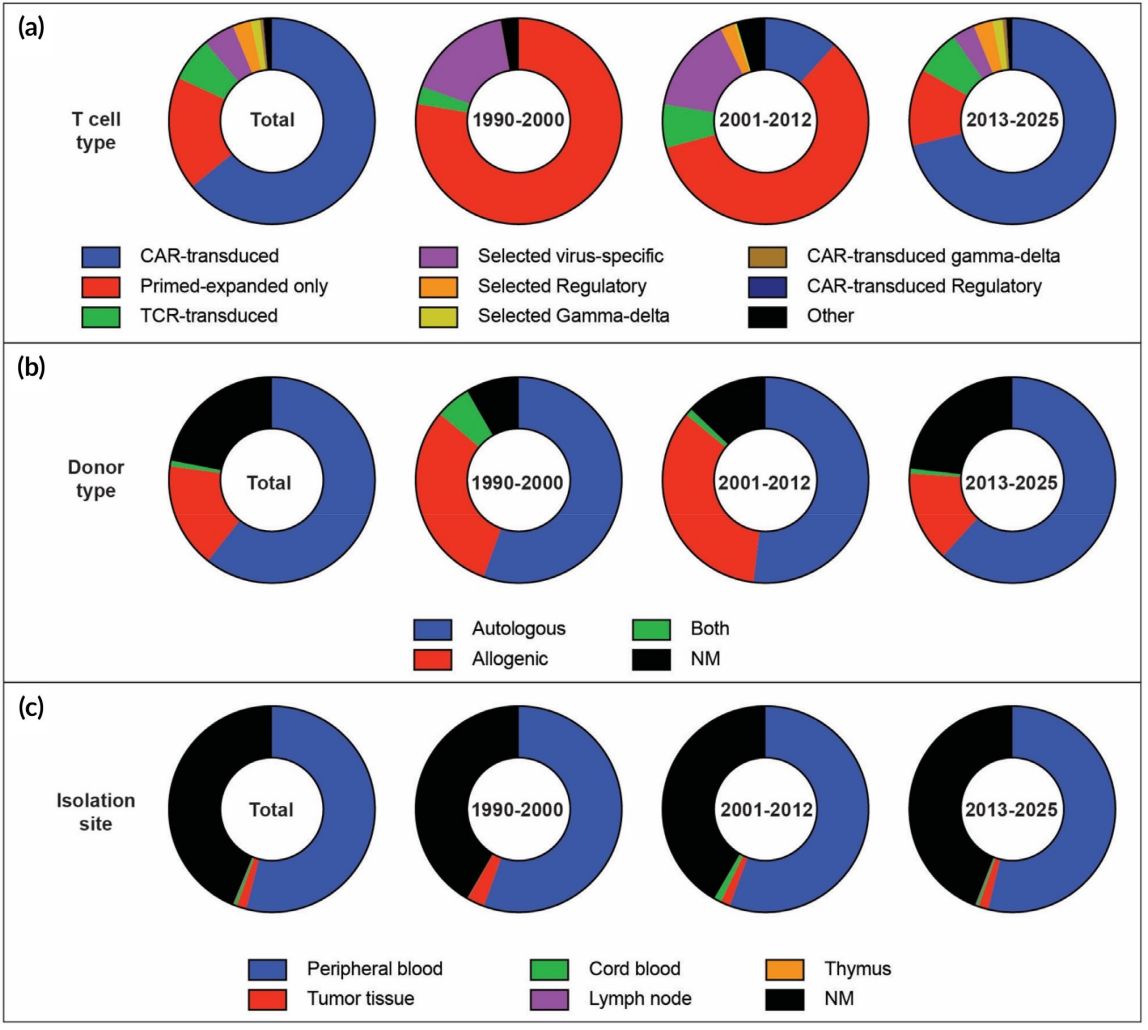
The landscape of T cell therapy clinical trials: Cell type and sourcing. (a)–(c) Distribution of trial specification dictated by cell type and sourcing across all stages and individual intervals: (a) T cell type, (b) donor type, (c) isolation site, NM, not mentioned.

A previous analysis of active clinical trials conducted by our group in 2021 found that the type of T cells used as an intervention strongly influences the disease indication investigated in clinical trials.^5^ Specifically, 98.6% of CAR T cell therapies were focused on cancer, with the majority targeting liquid tumors, while 90.7% of TCR‐transduced T cell therapies also targeted cancer but were more commonly directed toward solid tumors. In contrast, virus‐specific T cells were primarily evaluated for their role in treating transplant‐related complications and infections. Given that CAR T cells are currently the most favored cell type and all the currently approved CAR T cell products have liquid cancers as an indication, it is not surprising that 49.4% of all clinical trials focus on liquid cancers. However, this was not always the case. The rise of CAR T cells and their success in treating blood cancer have gradually shifted the clinical landscape toward blood cancer indications. Notably, in the 1990–2000 period, only 27.8% of trials focused on blood cancers. This proportion increased slightly to 34.8% in 2001–2012, and then significantly rose to 51.5% in the 2013–2025 interval, highlighting the influence of CAR T cell success on target indication (Figure 5a). In contrast, solid tumors were the primary focus in earlier years, with 47.2% of trials in 1990–2000 and 38.6% in 2001–2012, before declining to 33.4% in 2013–2025. The current prevalence of approved T cell products, coupled with their remarkable clinical success, has created a highly competitive landscape for liquid tumor indications. In contrast, numerous challenges have hindered the progress of T‐cell therapy in solid tumors.^63^, ^132^, ^133^ However, the recent approval of a few T cell therapies for solid tumors, which account for the majority of cancer‐related deaths, marks a potential strategic shift. These advances may likely catalyze developments and drive increased focus and investigation into solid tumor therapies, potentially shifting momentum away from the liquid cancer space in the coming years.^96^

In non‐cancerous diseases, the share of infectious diseases and transplant‐related disorders in T cell therapy trials has been relatively comparable, accounting for 4.3% and 4.5% of trials, respectively. However, both indications have experienced a decline over time. For infectious diseases, the proportion of T cell trials decreased from 19.4% in 1990–2000, to 11.7% in 2001–2012, and further to 3.2% in 2013–2025. A similar trend was observed in transplant‐related disorders, where trial representation fell from 8.3% in 2000–2012 to 4.1% in 2013–2025. This downward trend may reflect the growing promise of T cell therapies in cancer, which continues to pose significant therapeutic challenges, as well as the advancements in the management of infectious and transplant‐related conditions through alternative treatment strategies.^10^, ^127^, ^134^ Notably, the first trial using CAR T cells was initiated in 2000 for patients with HIV infection,^135^ at a time when the average life expectancy for HIV patients was just 36.1 years.^136^ Since then, advances in antiviral therapies have dramatically improved clinical outcomes, with life expectancy for HIV patients now approaching that of the general population,^137^ reducing the motivation for T cell‐based interventions in this area. On the other hand, autoimmune diseases, which represent 6.3% of all T cell therapy trials, have shown a renewed focus in recent years, much of which has occurred within the past 5 years. This resurgence is largely driven by the exploration of CD19 CAR T cells for treating autoimmune conditions, an approach that has shown remarkable early promise. Initially, the focus on autoimmune indications declined, with trial representation dropping from 5.6% in 1990–2000 to 2.7% in 2001–2012, similar to the trends seen in infectious and transplant‐related disorders. However, between 2013 and 2025, the share of trials targeting autoimmune diseases rose significantly to 6.8%. In our prior analysis of ongoing trials conducted in 2021, only 1.5% of T cell therapy trials focused on autoimmune diseases, a number that aligned with earlier trends of decline.^5^ This highlights the limited attention this area had received up to that point. However, 2021 marked a pivotal inflection point, which transpired with a notable case report demonstrating the remarkable clinical efficacy of CD19 CAR T cells in a life‐threatening case of SLE.^138^ With the chronic burden and initial remarkable success of T cell therapies in autoimmune diseases, these findings suggest that T cell therapies may offer a transformative approach, positioning autoimmunity as one of the most promising areas for exploration beyond oncology.^95^

Unsurprisingly, the blood and lymphatic system has emerged as the most commonly targeted pathological site in T cell therapy trials, accounting for 55% of all studies. This proportion has steadily increased over time, from 38.9% in 1990–2000 to 44.7% in 2001–2012, and further to 56.5% in 2013–2025 (Figure 5b). With the growing application of T cell therapies in systemic autoimmune diseases and hematologic malignancies, including T cell blood cancers,^95^, ^139^, ^140^ this upward trend can be expected to continue in the coming years. Trials targeting multiple solid tumors have shown a slight decline over time, decreasing from 30.6% in 1990–2000 to 24.2% in 2001–2012, and further to 21.7% in 2013–2025. Despite this decrease, such trials continue to be actively initiated, largely due to the prevalence of shared tumor‐associated antigens across various cancer types.^141^ These studies typically focus on broadly expressed targets such as Mesothelin (NCT06248697), KRAS (NCT06218914), HER2 (NCT01935843, NCT06658951), EGFR (NCT06682793), HPV (NCT06505551), B7‐H3 (NCT06612645), CEA (NCT05538195), among others. This strategy supports the development of multi‐indication therapies with the potential for broader clinical impact. Interestingly, the proportion of trials focused exclusively on skin cancers and related diseases has declined significantly over time, remaining relatively stable at 13.8% in 1990–2000 and 10.6% in 2001–2012, before dropping sharply to just 1.5% in 2013–2025. This trend may, in part, reflect the success of immune checkpoint inhibitors over the past decade, which have dramatically improved outcomes for patients with advanced skin cancers, including overall 5‐year survival rates exceeding 50% in unresectable cases.^142^ However, with the recent approval of a T cell therapy product for melanoma, there is potential for a resurgence of interest in T cell‐based approaches for skin cancer in the coming years, particularly in areas where resistance emerges with current therapies.

### 4.7 Delivery route

Given that all approved T cell therapies are administered intravenously, and the majority of these therapies target blood‐borne malignancies, it is not surprising that the intravenous (IV) route remains the predominant method in clinical investigations, reported in 91.4% of trials. This trend has remained consistent across time, with 86.1% of trials in 1990–2000, 88.6% in 2001–2012, and 91.8% in 2013–2025 utilizing IV administration (Figure 5c). In blood‐related malignancies, this preference for IV administration in T cell therapies stems largely from their mechanism of action, which relies on direct cell–cell contact to search and elicit effector activity.^143^ IV delivery allows for immediate systemic distribution, granting efficient access to malignant cells that typically reside within the peripheral blood compartment in such malignancies. For solid tissue‐related pathologies, it also remained the most widely accepted clinical route, both logistically and from a regulatory standpoint.^107^ However, growing clinical experience in solid tissue‐related pathologies, particularly solid tumors, has revealed the challenges of this approach in such cases. T cells administered intravenously face significant barriers to effectively reaching and infiltrating solid target sites.^144^ As a result, even with multiple infusions that extend T cell persistence in circulation, the therapeutic efficacy of systemically delivered T cells often remains suboptimal in the context of solid tumors.^96^ This growing understanding has prompted a slow but steady shift toward locoregional delivery routes for solid tumors, particularly for pathologies confined to specific anatomical sites, such as brain tumors.^145^ While no clinical trials reported the use of locoregional T cell delivery before 2000, 1.5% of trials between 2000 and 2012 adopted this approach. This has further increased to 2.5% of trials from 2013 to 2025, reflecting a gradual but notable rise in interest. Additionally, 0.6% of trials in the 2013–2025 period reported the use of intraperitoneal delivery, in part locoregional in nature, which had not been observed in earlier timeframes. A recent interim report from a phase 1 clinical trial (NCT02208362) evaluating locally administered CAR T cells through intrathecal infusion for the treatment of malignant gliomas demonstrated tumor reduction in all six patients, with substantial regression observed within just 24–48 h post‐infusion, even in the absence of lymphodepleting chemotherapy.^146^ In another example, a Phase 1 clinical trial (NCT02414269) utilizing local CAR T cell administration with intrapleural infusion reported a 1‐year overall survival rate of 83% and a median overall survival of 23.8 months.^147^ While these findings are preliminary, they show remarkable promise. With the logistical and clinical implementation of locoregional delivery becoming increasingly feasible,^107^ these trends underscore a growing effort to overcome delivery barriers in solid tumors through locoregional administration strategies. These locoregional delivery routes, including intraventricular, intrapleural, intraperitoneal, and intratumoral administration, help circumvent many of the barriers associated with IV delivery, while also holding the potential to elicit abscopal therapeutic effects.^148^ While no current T cell therapy trials employ biomaterial scaffolds for T cell delivery, preclinical studies have demonstrated that such approaches can yield strong abscopal effects, enabling the targeting of malignant cells at distant sites from the administration point. Additionally, some of these strategies have shown the ability to promote antigen spreading against non‐targeted tumor‐associated antigens and enhance T cell persistence and memory formation.^107^, ^149^ These findings offer compelling promise and support the investigation of biomaterial‐integrated T cell therapies in future trials.

### 4.8 Ex vivo modification

**FIGURE 6:**
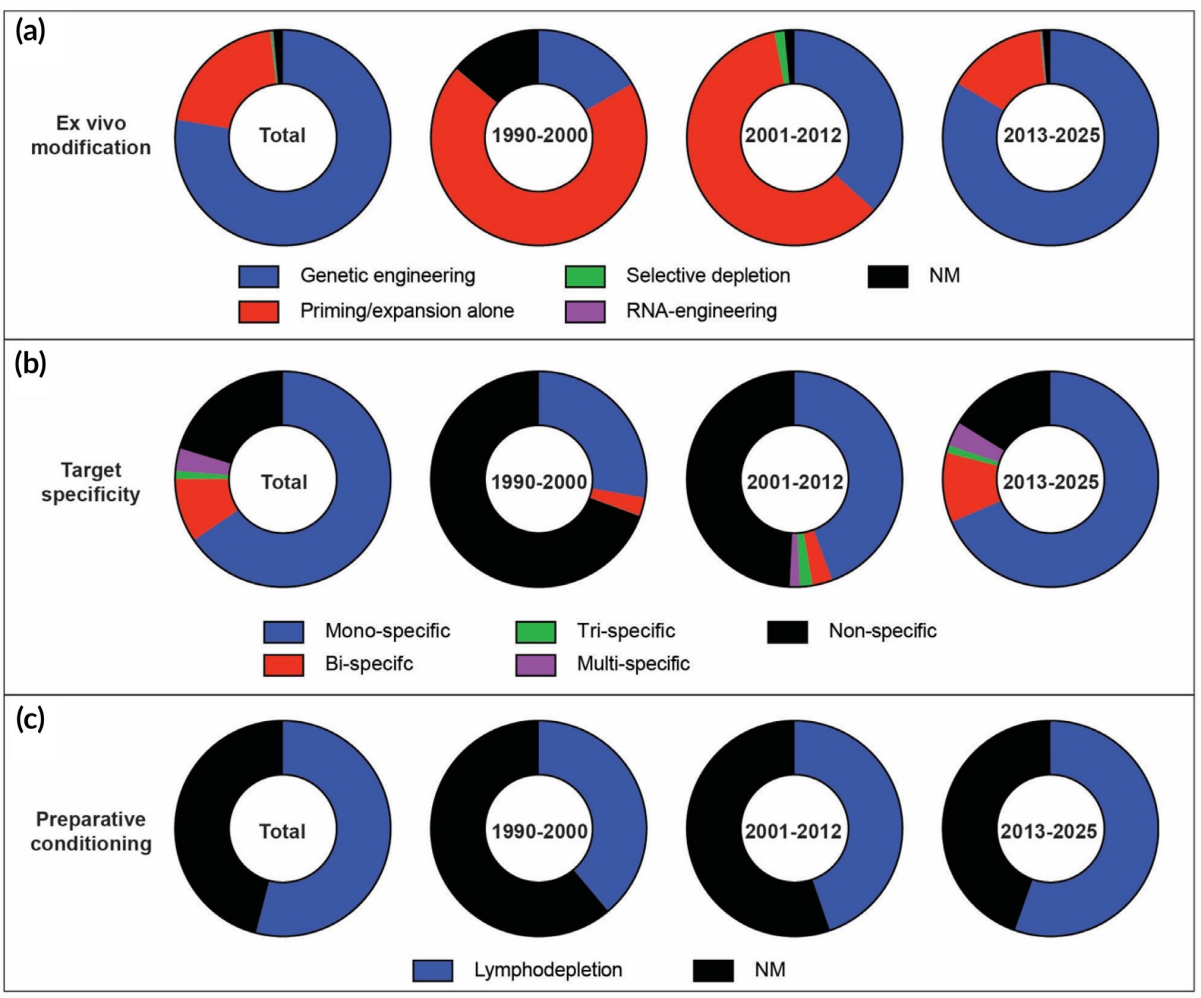
The landscape of T cell therapy clinical trials: Modifications and conditioning. (a)–(c) Distribution of trial specification dictated by ex vivo modification and conditioning across all stages and individual intervals: (a) Ex vivo modification, (b) target specificity, (c) preparative conditioning, NM, not mentioned.

T cell therapies typically require the infusion of approximately 1–100 billion T cells for therapeutic dosing.^150^ Given the scarcity of naturally occurring antigen‐specific T cells,^151^ the use of ex vivo rapid expansion and genetic modification protocols has become essential to generate sufficient numbers of therapeutic T cells capable of achieving effective dosing. In the 1990s, when genetic engineering was still in its early stages, the majority of clinical trials focused on priming and expanding T cells ex vivo without additional modifications. During 1990–2000, 69.4% of trials reported this approach, marking a period dominated by the development of REPs. However, as gene‐editing technologies matured, there was a significant rise in trials incorporating genetic engineering steps. The proportion of trials employing it increased from 16.7% in 1990–2000 to 36.7% in 2001–2012 and then surged to 83.4% in 2013–2025. This shift was accompanied by a marked decline in trials utilizing only priming and expansion protocols, dropping from 60.2% in 2001–2012 to just 15% in 2013–2025. Overall, 77.7% of clinical trials have incorporated genetic engineering modifications into their T cell manufacturing processes, and this trend is expected to remain a cornerstone of clinical development moving forward (Figure 6a). The initial clinical application of gene‐modified T cells can be traced back to the early 1990s when the Rosenberg group reported the adoptive transfer of TILs transduced with a retroviral vector encoding neomycin resistance in melanoma patients, establishing the safety and feasibility of gene transduction in a clinical setting.^56^ By 2000, the field advanced further with the first clinical investigation utilizing adoptively transferred T cells engineered with CARs via retroviral transduction in patients with HIV infection.^135^, ^152^ Despite these early milestones, genetic engineering did not become a mainstay of adoptive cell therapy until the early 2010s, when CAR T cells engineered with lentiviral^153^ or retroviral vectors^75^ demonstrated robust and durable anti‐tumor responses in clinical trials for hematologic malignancies (NCT00924326, NCT01029366). These pivotal results marked a paradigm shift, firmly establishing genetic engineering as a central strategy in adoptive T cell therapy. In 2020, the first clinical report using CRISPR‐Cas9 to edit PD‐1 genes via electroporation demonstrated the safety and feasibility of this approach in lung cancer patients (NCT02793856), introducing a powerful new tool to the genetic engineering toolbox in adoptive T cell therapy.^154^ Additionally, the newly emerging field of gamma‐delta T cell therapies, which initially relied solely on priming and expansion, has now also begun to incorporate genetic engineering approaches. Notably, 41.3% of trials involving gamma‐delta T cells now employ genetic modification, with the vast majority of these trials initiated within the past few years. A similar, albeit slower, shift is observed in regulatory T cell therapies, which have traditionally relied on selective enrichment, priming, and expansion. Currently, 11.7% of these trials incorporate genetic engineering, with the vast majority initiated within the past few years. This trend reflects the growing reliance on genetically modified T cell products to enhance specificity, persistence, and therapeutic efficacy. Overall, popular viral vectors, which are conventionally used for T cell genetic editing, continue to raise concerns among regulatory agencies due to the potential delayed risk of adverse events, including insertional mutagenesis. As a result, non‐integrating vector systems have become increasingly desirable, as they reduce the risk of insertional mutations and the potential for secondary malignancies associated with conventional viral vectors.^155^ In this context, the recent success of RNA‐based therapeutics, particularly during the global mass vaccination efforts in 2020, has sparked growing interest in applying RNA technologies to overcome key limitations of conventional genetic engineering in T cells.^156^ RNA‐based approaches aim to increase the efficiency of transduction, reduce the risk of tumorigenicity associated with permanent genomic integration, and avoid runaway toxic effects while achieving therapeutic efficacy.^157^, ^158^ No RNA‐based engineering approaches were reported in T cell therapy trials during the 1990–2000 and 2001–2012 intervals. A pivotal study published in 2014^159^ demonstrated the feasibility and safety of engineering T cells with mRNA targeting mesothelin, showing no apparent toxicity to normal tissues, supporting the clinical potential of RNA‐based strategies (NCT01355965). In the 2013–2025 period, 0.22% of trials employed RNA engineering technologies for T cell modifications. Notably, the majority of these trials were initiated after 2020, with most targeting BCMA. This marks an exciting advancement in the field of adoptive T cell therapy. Engineering cells with mRNA enables faster and more affordable manufacturing, while allowing transient, controlled gene expression without permanent integration of transgenes.^160^ As these trials progress, it will be interesting to observe whether RNA‐based approaches offer advantages over traditional genetic engineering and whether their adoption continues to grow in the coming years. Furthermore, strategies to engineer T cells directly in vivo are being developed to overcome the biological complexities and logistical challenges of ex vivo manufacturing, though these efforts are still in their early stages.^161^ These developments have the potential to mark a new inflection point in the evolution of T cell therapies, improving global accessibility and affordability while simultaneously reducing safety concerns without compromising therapeutic outcomes associated with conventional approaches.

### 4.9 Target Specificity

The primary reason for the dominance and clinical success of T cells over other immune cell types in adoptive therapies lies in their ability to restrict effector activity to target‐specific antigens.^5^ This high degree of precision holds a key to not only enhancing safety but also ensuring a potent and effective therapeutic response.^162^ As a result, target specificity plays a critical role in the design of T cell therapies, a point supported well by the fact that 79.6% of all T cell therapy trials have a specified target antigen. Notably, the focus on defined targets has grown significantly over time. While only 30.6% of T cell therapy trials between 1990 and 2000 reported a specific target, this figure rose to 50.8% in 2001–2012 and further increased to 83.8% in the 2013–2025 period (Figure 6b). Among these trials, the majority are monospecific, with 65.4% of all trials focusing on a single target. However, tumor relapse and disease progression due to antigen escape, particularly in hematologic malignancies where early responses to therapy are often strong, along with the inability to mount an effective response in antigen‐heterogeneous solid tumors, have prompted a shift toward multi‐targeted strategies.^163^ This shift toward multi‐targeted approaches did not gain significant momentum until extensive clinical experience was accumulated with monospecific strategies, and advancements in genetic engineering made it feasible to design T cells capable of recognizing multiple antigens. In the 1990–2000 period, only 2.8% of trials explored bi‐specific targeting, with a modest increase to 3.0% in 2001–2012. However, in the 2013–2025 period, this number has risen more substantially to 10.5% of trials, reflecting a growing recognition of the need to address antigen escape and tumor heterogeneity in both hematologic and solid malignancies. A prominent example of the limitations of monospecific targeting can be seen from the experience of clinically approved CAR T cell therapy approaches, especially in multiple myeloma.^164^ Clinical trials (NCT02215967, NCT02658929) investigating monospecific CAR T cell therapies targeting BCMA have revolutionized treatment in this indication, leading to the approval of multiple products.^165^ However, relapse due to BCMA‐negative antigen escape has been increasingly reported following treatment, highlighting a major challenge and leaving the disease incurable in many cases. In response, additional targets such as GPRC5D have gained attention,^166^ with monospecific CAR T cells targeting GPRC5D now being evaluated in a Phase 3 clinical trial (NCT06615479). However, a recent study highlights the limitations of monospecific targeting by showing that only 65% of patient samples had GPRC5D expression above the threshold, while 73% met the threshold for BCMA. These findings indicate that both targets, when used individually, carry a risk of antigen escape. Interestingly, when combining both targets, 88% of patient samples met the expression threshold,^166^ suggesting that a bispecific approach targeting both BCMA and GPRC5D could be significantly more effective and durable than either monospecific strategy alone. This idea is further supported by a recent preclinical study^167^ prompting multiple clinical trials (NCT05509530, NCT06793475, and NCT06655519). Similar strategies are being pursued to address relapse in another clinically successful monospecific approach, CAR T cells targeting CD19.^168^, ^169^ To overcome antigen escape and improve response durability, bispecific CAR T cell therapies targeting CD19 and CD22 (NCT06081478, NCT06078306) or CD19 and CD20 (NCT06009107, NCT05990465) are currently under clinical investigation. These trials aim to deliver more robust and sustained clinical responses by simultaneously targeting multiple B‐cell antigens. The challenge of antigen heterogeneity and associated therapeutic resistance is increasingly prompting the development of multi‐antigen targeting strategies, especially in solid tumors.^170^ As a result, clinical trials are now exploring tri‐specific (NCT04637503, NCT04430595, and NCT02287311), quad‐specific (NCT05768880), penta‐specific (NCT05471661), and tumor‐associated multispecific T cell therapies, intending to enhance efficacy and minimize the risk of antigen escape. The proportion of clinical trials investigating such multi‐antigen targeting approaches has increased from 3.4% in 2000–2012 to 4.5% in 2013–2025, and this upward trend is expected to continue as cell editing technologies advance in precision and efficiency.^17^ This evolving strategy holds the potential to overcome tumor heterogeneity and resistance mechanisms, paving the way for more durable and broadly effective T cell therapies. However, the gradual diversification of malignant cells during tumorigenesis and subsequent metastasis, accompanied by infiltration of diverse immune and stromal cells, introduces biological, spatial, and temporal antigenic variability, which can lead to antigen escape. This phenomenon may be further exacerbated by immune pressure exerted by therapeutic cytotoxic T cells, which can actively shape the tumor microenvironment and promote immune evasion. As a result, simply targeting multiple antigens simultaneously may be insufficient.^171^ A more effective strategy would involve enhancing immunogenic cell death and promoting epitope spreading, thereby initiating a broader endogenous immune response capable of adapting to tumor heterogeneity.

### 4.10 Preparative conditioning

Preparative conditioning regimens originated from clinical experience with HSCT. Initially, high‐dose chemotherapy and radiation were used to treat malignant diseases but were found to cause bone marrow ablation and irreversible cytopenia, necessitating HSCT for hematopoietic recovery. Over time, allogeneic HSCT was recognized not only for its regenerative role in immune reconstitution but also for its therapeutic potential in eliminating residual disease. As a result, conditioning regimens evolved into supplementary preparative protocols designed to create space for donor stem cell engraftment, reduce the risk of graft rejection, and ultimately enhance the durability of therapeutic responses.^172^ These benefits seen in the HSCT prompted efforts to incorporate conditioning regimens that deplete endogenous B cells, T cells, Tregs, and natural killer (NK) cells to create a lymphodepleted environment prior to adoptive T cell therapy.^173^ These lymphodepleting regimens, most commonly consisting of cyclophosphamide and fludarabine, were shown to enhance the proliferation and persistence of transferred T cells by reducing competition from the host lymphocyte pool and eliminating cytokine sinks that otherwise consume homeostatic and T cell–supportive cytokines such as IL‐2, IL‐7, and IL15.^174^ The use of lymphodepleting chemotherapy in clinical trials has steadily increased over time, rising from 38.9% in 1990–2000 to 44.7% in 2001–2012, and reaching 55.4% in the 2013–2025 period (Figure 6c). This trend is even more pronounced in approved T‐cell therapy products, with 89.5% incorporating lymphodepleting chemotherapy as part of the treatment protocol. Overall, 54.1% of T cell therapy trials report the use of preconditioning lymphodepleting chemotherapy. This approach is most prevalent in blood cancer trials, where 60% of trials report lymphodepletion. In contrast, only 44.8% of T cell therapy trials targeting autoimmune diseases report the use of lymphodepleting regimens. This difference likely reflects the distinct therapeutic strategies employed: while most T cell therapies in blood cancer aim to eliminate pathological cells, certain approaches in autoimmune diseases, particularly those involving Tregs, focus on inducing immune tolerance and often do not necessitate a conditioning regimen.^123^, ^175^ Such preparative lymphodepletion approaches have also been extended to the treatment of solid tumors, with 56.4% of trials reporting their use. However, in the context of brain tumors, where T cell therapies are frequently administered via locoregional routes, systemic lymphodepletion is often considered less critical.^145^, ^176^ As a result, only 39.5% of brain tumor trials report the use of preparative lymphodepleting chemotherapy. These trends indicate that while lymphodepleting conditioning regimens have been essential to the success of T cell therapies, their associated toxicity necessitates^65^ careful consideration of their use based on the specific mechanism of action of the T cell therapies.

## 5 CHALLENGES IN CLINICAL TRANSLATION

As T cell therapies expand into newer indications and more diverse patient populations, a range of scientific and technological challenges has emerged that hinders their broader clinical implementation. These challenges can be broadly categorized into scientific hurdles, such as functional decline, trafficking inefficiencies, and target heterogeneity, and technological constraints, including cell sourcing, manufacturing complexity, and scalability. Addressing these hurdles is critical for the successful and widespread clinical translation of T cell therapies. In this section, we discuss these key translational barriers and highlight notable efforts aimed at overcoming them.

### 5.1 Scientific hurdles

**FIGURE 7:**
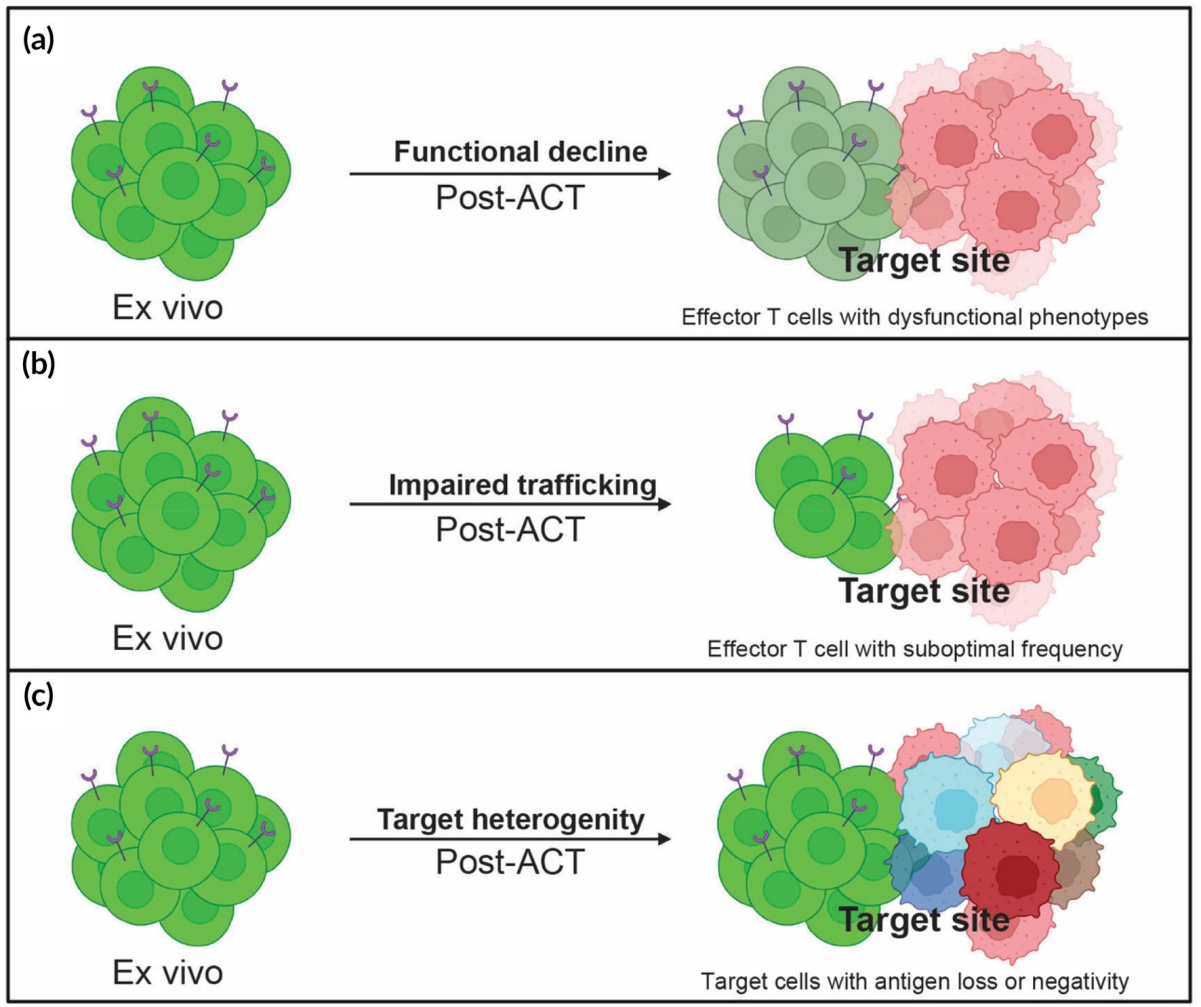
Scientific hurdles limiting the clinical effectiveness of T cell therapies. This figure illustrates the key biological challenges that T cells (green) encounter post‐transfer, depicted here as three distinct categories for visual clarity. In clinical practice, these challenges often occur simultaneously at the target site with malignant cells (red), with varying degrees of impact depending on the pathological context and design strategy of the T cell therapy. (i) Functional decline: Despite optimal ex vivo manufacturing, T cells frequently experience a decline in effector function after adoptive transfer. Dysfunctional T cells are often identified at the target site, exhibiting reduced cytokine secretion, cytotoxicity, and persistence.^182^ In the figure, functional decline is represented by a faded saturation of the green color in T cells at the target site. (ii) Impaired trafficking: The ability of infused T cells to accumulate in sufficient numbers at the disease site is frequently inadequate to counteract pathological progression.^221^ This challenge is depicted by the low number of T cells observed at the target site in the illustration. (iii) Target heterogeneity: T cell therapies often rely on a narrow antigen recognition repertoire, targeting only a limited set of target‐associated antigens.^204^ This selective pressure can drive immune editing, resulting in the emergence of antigen‐negative malignant cells that evade T cell recognition and contribute to relapse. In the figure, antigen escape is represented by a heterogeneous population of malignant cells (shown in various colors) at the target site. Created with BioRender.

Several key challenges have emerged following adoptive T cell transfer, primarily related to the biological properties of the T cells themselves. The most prominent issues include diminished effector function, inefficient trafficking to the target site, and antigenic heterogeneity within the target site. For clarity, Figure 7 presents these challenges as distinct categories, but in practice, they often occur simultaneously and to varying extents, depending on the design strategy of the T cell therapy and the pathological context in which it is applied.

#### 5.1.1 Functional decline

In many disease settings where T cell therapies are applied, endogenous T cells are frequently observed to be dysfunctional, largely due to unsuitable microenvironments shaped by pathological changes.^177^ For example, hypoxia and low nutrient levels limit the function of cytotoxic T cells in solid tumors,^178^ inhibitory checkpoint signaling suppresses virus‐specific T cells during chronic infections,^179^ and pro‐inflammatory conditions disrupt Treg function in autoimmune diseases.^180^ These pathology‐induced factors not only compromise endogenous T cells but are also found to adversely affect adoptively transferred T cells post‐infusion. Therefore, the therapeutic efficacy of T cell therapies depends on the ability of transferred T cells to persist and retain effector functionality within these challenging in vivo environments. A clinical investigation of adoptive cell therapy using in vitro expanded TILs in advanced melanoma (NCT03475134) performed a dynamic analysis of TIL states, tracking their progression from baseline tumor samples at the time of harvesting, through the manufactured ACT product, and finally to post‐infusion tumor samples. Patients with enriched tumor‐reactive TILs demonstrated clinical responses and exhibited baseline TILs with an exhausted transcriptional profile that was functionally reinvigorated during ex vivo expansion. However, by 30 days post‐transfer, these TILs reacquired exhaustion‐associated features, highlighting the challenge of sustaining T cell functionality in vivo to achieve durable therapeutic responses.^181^ While T cell dysfunction has traditionally been attributed to persistent antigen exposure and an immunosuppressive microenvironment over days to weeks,^178^ recent preclinical studies in solid tumors suggest that the decline in T cell functionality begins much more rapidly within hours post‐transfer, even before the first cell division occurs.^182^ Notably, this rapid functional impairment is observed even in T cells that are robustly primed and expanded ex vivo. The sustained adverse in vivo environment contributes to a progressively dysfunctional state, reinforcing early hallmarks of T cell exhaustion. The antigen‐recognition domains introduced via ex vivo genetic engineering have also been shown to govern T cell functional decline. In the case of CAR T cells, CAR domain clustering can induce spontaneous tonic signaling, which significantly impacts T cell fitness and persistence, including CTLs and Tregs.^183^ For example, one study found that CAR constructs targeting GD2 and CSPG4 generate high levels of tonic signaling, promoting T cell exhaustion and reduced efficacy. However, modulating tonic signaling down to an optimal threshold improved both persistence and therapeutic response post‐adoptive transfer. Conversely, CARs targeting CD19 and CD22, which exhibit low tonic signaling, showed enhanced post‐transfer persistence when tonic signaling was increased to a moderate level.^184^ Furthermore, differences in the quality and composition of T cells within infusion products have been shown to significantly impact post‐transfer persistence and therapeutic efficacy.^185^, ^186^ In a real‐world analysis of clinically approved CAR T cell therapies, such as Kymriah and Yescarta, the presence of less‐differentiated central memory T cell phenotypes in the infused product correlated with enhanced in vivo persistence and greater expansion of T cells following adoptive cell transfer.^187^ These findings underscore the importance of T‐cell functional persistence as a key determinant of treatment durability and clinical response.

Strategies to mitigate functional decline in T cells can be broadly categorized into three approaches: (i) Ex vivo differentiation of T cells into phenotypes that demonstrate improved fitness and persistence, (ii) Co‐delivery of supportive agents alongside T cells to enhance their function and survival post‐infusion, (iii) Genetic engineering of T cells with armored constructs, enabling the co‐expression of synergistic molecules in addition to the primary antigen‐recognition domain. Ex vivo differentiation strategies aim to prime and expand T cells while preserving less‐differentiated, memory‐like phenotypes, using culture media supplemented with homeostatic cytokines, epigenetic modulators, or tonic signaling inhibitors.^188^, ^189^ Several clinical trials have been initiated using T cell products cultured in media containing cytokines such as IL‐15 (NCT05359211, NCT05103631, NCT04715191, NCT04377932, NCT03721068, and NCT02992834) and small molecules like PI3K inhibitors (NCT03274219), which have shown preclinical promise in enhancing memory phenotype and improving T cell persistence post‐transfer. However, these effects are often transient, especially in solid tumors, and T cells tend to differentiate into less functional states following infusion.^96^ In co‐delivery approaches, supportive agents are administered alongside T cells to maintain their in vivo activity post‐transfer. These strategies include the delivery of free cytokines such as IL‐2 (NCT03215810, NCT01493453), IL‐15 (NCT01369888), and IL‐15 receptor agonists (NCT05359211); membrane‐conjugated cytokines, such as IL‐12 (NCT06474676, NCT05621668); and membrane‐anchored nanoparticles loaded with immunostimulatory agents, including IL‐15 superagonist (IL‐15sa) (NCT03815682) and IL‐12 (NCT04762225). These adjuncts aim to support T cell persistence, proliferation, and effector function in the hostile in vivo microenvironment.^149^ The differential pharmacokinetics between supportive agents and T cells presents a key limitation to these co‐delivery strategies, often resulting in rapid clearance of the agents and untargeted systemic distribution. To overcome these challenges, ongoing efforts are focusing on biomaterial‐based delivery systems that enable controlled release and spatially coordinated delivery of supportive cargos.^148^, ^149^, ^190^, ^191^ These biomaterial integration strategies are being explored at various scales, from nanoparticle carriers to scaffold‐based platforms. Genetic engineering approaches are evolving beyond conventional antigen‐recognition domains, enabling the co‐expression of additional molecules that are either secreted by T cells or upregulated on their surface to enhance T cell function and persistence post‐transfer.^192^ The second‐generation CAR constructs, which added a co‐stimulatory domain, showed improved persistence and led to the clinical approval of these CAR T cell therapies in blood cancers. However, their efficacy in solid tumors has remained modest. To address these limitations, third‐generation CARs, with multiple co‐stimulatory domains, have been developed to enhance durability. Additionally, fourth‐generation CARs incorporate inducible expression of stimulatory agents such as cytokines, while fifth‐generation CARs include additional membrane‐bound receptors to further reinforce T cell functionality within the tumor microenvironment.^193^ Clinical trials are currently investigating these advanced genetic engineering strategies, including CAR T cells engineered to secrete cytokines such as IL‐12 (NCT02498912, NCT06343376), IL‐15 (NCT03721068, NCT05103631), TEAM (NCT05660369), and IL‐18 (NCT04684563), as well as those designed to upregulate IL‐2 receptor expression (NCT05665062). These genetic modifications aim to enhance T cell persistence and functionality in vivo. While early results are promising, the constitutive secretion of immunostimulatory molecules also poses a potential risk of systemic toxicity and runaway reactions. To address these concerns, ongoing efforts are focused on developing controlled expression systems, including logic‐gated circuits and triggered release mechanisms, to enable context‐dependent expression and improve safety without compromising efficacy.^96^

#### 5.1.2 Impaired trafficking

As identified earlier, the IV route remains the predominant method for administering T cells. While this route provides direct access to malignant cells in hematologic compartments, it poses significant challenges when targeting solid tissues, where T cells must navigate a series of physical and biochemical barriers to exert their effector function.^143^ These barriers include limited chemokine‐mediated recruitment, poor vasculature maturity, and a dense extracellular matrix, all of which hinder effective T cell infiltration into solid tissues. Although T cells are generally more capable of trafficking through these barriers compared to conventional targeted therapies, including their ability to cross the blood–brain barrier,^4^, ^5^ their trafficking efficiency remains insufficient to mount a robust effector response in many solid pathological conditions. Impaired trafficking can lead to broader distribution away from the intended target site, thereby increasing the risk of off‐target activity and associated toxicities. As a result, there is often a failure to achieve adequate T cell accumulation at the target site, which is necessary to counteract rapidly progressing pathological disease.^96^ Achieving sufficient accumulation of T cells at the target site is critical for enhancing the safety and efficacy of T cell therapies.

Strategies to mitigate impaired T cell trafficking can be broadly categorized into three key approaches: (i) Locoregional delivery of T cells to bypass the physical and biochemical barriers associated with systemic administration; (ii) Repeated infusions to maintain sufficient T cell levels at the target site over time; and (iii) Genetic engineering of T cells with armored constructs that co‐express migration‐enhancing molecules in addition to the primary antigen‐recognition domain. Our clinical trial analysis indicates a growing interest in locoregional T cell delivery, particularly for diseases that are anatomically confined. Several ongoing trials are investigating the feasibility and therapeutic benefit of this approach, including intraventricular, intrathecal, or intracerebroventricular delivery for central nervous system (CNS)‐related diseases (NCT05660369, NCT05168423, and NCT04196413), intrapleural delivery for malignant pleural diseases (NCT02414269), and intraperitoneal delivery for diseases within the peritoneal cavity (NCT06623396, NCT06215950, and NCT03585764). While locoregional administration holds promise for enhancing local T cell concentration and improving outcomes in site‐restricted diseases, local biological barriers at the target site, such as immunosuppressive microenvironments and poor tissue penetration, continue to limit therapeutic efficacy even after direct delivery.^107^ Multiple infusion strategies are increasingly being adopted as an alternative to single‐dose interventions, aiming to ensure adequate accumulation of transferred T cells at the target site over time.^96^ Rather than relying on a one‐time infusion, T cells are administered in multiple doses at regular intervals to enhance therapeutic coverage and circumvent inefficient accumulation. Several ongoing clinical trials are exploring this approach, including three or more locoregional infusions for brain tumors (NCT02208362), three cycles of IV infusions for CNS malignancies (NCT03726515), and three IV administrations for gastrointestinal cancers (NCT03874897). These repeated dosing regimens have been shown to be feasible and safe, with early indications of improved therapeutic efficacy, particularly in achieving increased therapeutic T cell levels at the disease site.^194^, ^195^ An additional strategy to enhance T cell trafficking efficiency involves genetic engineering to upregulate migration‐enhancing molecules. These include adhesion molecules and their ligands to improve homing, chemokine receptors to better sense and respond to chemotactic gradients, and proteolytic enzymes to facilitate extracellular matrix degradation and tissue penetration. While many of these approaches have demonstrated promise in preclinical models, only a limited number have progressed into clinical evaluation. Notable examples include anti‐CD19 CAR T cells co‐expressing CCL9 and IL‐7 for B cell lymphoma (NCT03258047), anti‐EGFR CAR T cells engineered with CXCR5 for lung cancer (NCT05060796), anti‐BCMA CAR T cells expressing CXCR4 for multiple myeloma (NCT04727008), virus‐specific T cells expressing CXCR4 for HIV infection (NCT03020524), and CXCR2‐transduced TIL for advanced melanoma. However, the complexity of trafficking mechanisms combined with the potential risks associated with continuous or unregulated expression of these molecules necessitates careful consideration and control before widespread clinical translation.^96^, ^196^, ^197^

#### 5.1.3 Target heterogeneity

One of the key advantages of T cell therapies, their target‐specific therapeutic activity, is increasingly being recognized as a potential limitation for eliciting curable responses.^198^ In many diseases characterized by antigenic diversity, this high degree of specificity can lead to antigen loss or escape due to immune editing driven by the pressure exerted by the transferred T cells. As a result, tumors or diseased cells may downregulate or lose the targeted antigen, enabling escape from immune surveillance and contributing to treatment failure over time.^199^ To address these challenges, various strategies have been developed to circumvent narrow target specificity and instead promote polyclonal T cell responses post‐transfer. These approaches can be broadly categorized into three main areas: (i) engineering or culturing T cells to generate a diverse population with multi‐specific clonality, enabling recognition of multiple antigens; (ii) labeling or modifying the target site to present a homogeneous set of antigens, thereby enhancing recognition by transferred T cells; and (iii) combining synergistic immunomodulatory strategies to promote antigen spreading, thereby broadening the immune response and enabling polyclonal activation at the target site post‐transfer. As discussed in Section 4.7, there is a growing number of clinical trials investigating T cell therapies targeting multiple antigens, including the development of penta‐specific T cells. Several of these multi‐targeted approaches have advanced to late‐stage clinical trials, offering significant optimism in addressing challenges such as relapse and resistance‐related treatment failure. As the number of targets recognized by T cells increases, it becomes critically important to ensure that this targeting occurs in a controlled manner to maintain safety and minimize off‐target effects.^96^ The selection of an optimal combination of antigen candidates, those that are exclusively expressed on malignant cells and absent from healthy tissues, is critical for ensuring the specificity and safety of T cell therapies.^200^ However, truly tumor‐specific surface antigens are exceedingly rare and often change dynamically, especially in tumors with a low mutation burden, such as glioblastoma.^201^ Additionally, tumor heterogeneity further complicates the identification of such optimal antigen combinations. To mitigate the risk of on‐target, off‐tumor toxicity, several strategies have been explored, including the design of logic‐gated circuits, tuning TCR affinity, and incorporating exogenous control mechanisms or antigen density thresholds to refine activation and improve precision. On the other hand, rather than broadening T cell specificity, an alternative strategy involves inducing homogeneous expression of a single artificial antigen at the target site, thereby enabling a universal T cell therapy approach that is independent of the native antigenic diversity of the pathology. Examples of this approach include the use of anti‐CD19 CAR T cells following intratumoral administration of an oncolytic virus encoding CD19 in pancreatic cancer, a setting that lacks well‐defined tumor‐restricted antigens.^202^ Similarly, anti‐FITC CAR T cells have been employed after local delivery of a FITC‐labeled amphiphile to tumors with heterogeneous antigen profiles.^203^ While these methods offer the potential for creating universal targeting strategies that bypass the need for natural tumor antigens, their effectiveness could be limited by challenges in achieving efficient, consistent, and tumor‐specific delivery and expression of the artificial labeling molecules. Additionally, combinatorial strategies that promote antigen spreading are being developed to synergize with the initial potent cytotoxic activity of transferred T cells with a narrow antigenic repertoire.^204^ These approaches aim to induce a cascade of endogenous immune activation after the initial activity of transferred T cells, ultimately generating a robust polyclonal immune response capable of providing long‐term tumor control and preventing antigen escape. Numerous clinical investigations have been initiated to evaluate combinatorial strategies that promote antigen spreading and enhance the efficacy of T cell therapies. These include combinations with vaccines (NCT05801913, NCT05432635, and NCT03291444), radiotherapy (NCT05800405, NCT05805371, and NCT05336383) and CTLA‐4 checkpoint inhibitors (NCT04003649). While a broad range of combinatorial options exists, their diverse mechanisms of action and variable synergistic potential make it challenging to directly compare outcomes across studies. A key challenge remains in optimizing dosing schedules and sequencing strategies to achieve maximum therapeutic synergy while minimizing additive toxicities.^205^

### 5.2 Technological challenges

Several key technological and logistical challenges have emerged in the real‐world implementation of adoptive T cell therapies in clinical trials as well as commercially approved products, limiting their broader adoption across diverse settings and patient populations. Among the most significant barriers are cell sourcing constraints and complex, resource‐intensive manufacturing processes. These limitations hinder the scalability and accessibility of T cell therapies, not only within currently approved disease indications but also across broader healthcare systems. Addressing these challenges is critical to establishing T cell therapies as routine, globally accessible treatment options.

#### 5.2.1 Cell sourcing

T cell activity is tightly regulated, and the risk of alloreactivity due to mismatching makes autologous cell sourcing the preferred approach for most T cell therapies. The quality and quantity of isolated T cells play a critical role in determining the efficacy of adoptive transfer.^206^, ^207^ For genetically modified cells such as CAR T cells, most T cell therapies are currently administered as late‐line treatments, often in patients who have undergone extensive prior therapies, including chemotherapy. As a result, many patients present with cytopenia and poor‐quality T cells, leading to low cell yields and reduced product quality. Recent analyses have shown that chemotherapy exposure prior to leukapheresis can induce phenotypic shifts in T cells toward more differentiated and exhaustion‐prone states compared to treatment‐naive individuals.^208^ T cell products derived from pretreated patients consistently exhibit diminished functionality compared to treatment‐naive patients. Furthermore, manufacturing failure rates for autologous T cell products range from 1% to 13% across studies, leaving some patients without the opportunity to receive these therapies.^209^ In non‐modified approaches such as TIL therapy, challenges in obtaining sufficient tumor tissue and the limited presence of naturally occurring TIL populations in many tumors render a subset of patients ineligible for such therapies.^210^ The challenges associated with autologous T cell sourcing have prompted growing interest in alternative strategies, including the use of allogeneic T cells and iPSC‐derived T cell generation. While allogeneic T cells offer the potential for off‐the‐shelf universal therapies, they carry a risk of alloreactivity, which can lead to reduced persistence, graft‐versus‐host responses, and increased toxicity in patients.^211^ To mitigate these risks, several clinical trials are investigating engineered allogeneic T cell products with disrupted expression of key receptors involved in alloreactivity, such as HLA, TCR, and CD3ζ (NCT02746952, NCT04244656, and NCT04384393). A reduction in immunogenicity can help mitigate host‐derived immune responses that may lead to the elimination of the therapeutic product, and it may also enable multiple administrations over time. However, even minor antigenic differences, such as those arising from HLA polymorphisms or partially matched donors, can provoke immune reactions against engineered T cells. In particular, pre‐existing or treatment‐induced immune responses to gene constructs, such as murine‐derived single‐chain variable fragments, have been associated with treatment failure and limited success in redosing strategies in some patients. These immunogenic responses have also been observed in solid tumor cases, where immunogenicity‐related events are more prominent.^212^ Importantly, immunogenicity varies depending on the specific gene construct, patient immune profile, and malignancy type.^213^ These observations highlight the need to carefully balance the induction of a potent endogenous immune response against malignant cells while minimizing immune responses targeting transgenic, engineered T cells. This challenge highlights the importance of monitoring anti‐transgene immunity and conducting studies to assess immunogenicity risks, to ensure the durability, safety, and efficacy of engineered T cell therapies, particularly in the context of multiple dosing strategies.

In parallel, researchers are also exploring cell types with T cell‐like functions that operate in an MHC‐independent manner, thereby lowering the risk of alloreactivity.^3^, ^46^ This includes the use of allogeneic gamma‐delta T cells (NCT06279026, NCT06092047, and NCT06417398), NKT cells (NCT06251973), and NK cells (NCT01576692, NCT02845999, and NCT00536978) in adoptive cell therapies. These approaches hold promise for expanding access to universal, readily available cell therapy products with improved safety profiles and broader clinical applicability. Additionally, T cells derived from induced pluripotent stem cells (iPSCs) are being actively explored as an alternative source for adoptive cell therapies. iPSCs can be reprogrammed and differentiated into less‐differentiated T cells by using specific differentiation cocktails.^214^ Furthermore, HLA‐homozygous iPSC lines can be banked, enabling the creation of a library of matched HLA combinations that can be used to generate a broad spectrum of immunologically compatible products, thereby minimizing the risk of allo‐rejection.^215^ Clinical trials are currently underway to evaluate iPSC‐derived T cell products in indications such as hematologic malignancies (NCT04629729) and autoimmune diseases (NCT06308978). These efforts hold promise for producing scalable, off‐the‐shelf T cell therapies with reduced manufacturing costs and broader patient accessibility compared to traditional autologous approaches.

#### 5.2.2 Manufacturing protocol complexities

Ex vivo T cell therapy protocols involve a series of resource‐intensive steps, from T cell collection and manufacturing to post‐infusion patient management. This complexity contributes to high developmental costs and prolonged turnaround times, limiting access to these therapies even in well‐resourced, urban healthcare systems. Much of this complexity stems from the customized, patient‐specific nature of these products, with very limited availability of off‐the‐shelf options, leading to challenges in reproducibility and standardization. The commercial viability of such therapies depends not only on regulatory market authorization but also on insurance coverage and reimbursement criteria.^216^ Barriers such as the limited availability of comparative analysis of efficacy data, high failure rates due to manufacturing lapses and the possibility of delayed adverse events, particularly those with permanent genetic modifications, raise critical questions of long‐term clinical value. Additionally, instances where real‐world evidence does not fully support the therapeutic benefit post‐approval highlight the need for consistent design and manufacturing standards, especially given the high cost and anticipated curative potential of these one‐time treatments. Notably, the inability to meet national reimbursement requirements, such as those set by the European Union (EU), can lead to the market withdrawal of several approved products.^83^, ^216^ These challenges are even more pronounced in rare diseases, where small patient populations limit the ability to generate sufficient evidence to meet stringent standards for approval and reimbursement. Further, the current approved T cell therapies that rely heavily on centralized, industry‐driven manufacturing and logistics face significant real‐world implementation challenges, particularly in low‐ and middle‐income countries. It is estimated that only one in four patients registered for CAR T cell therapy ultimately receives the treatment, with a median wait time of up to 6 months for those who do, due to complex logistics, cost, and manufacturing limitations of T cell therapy.^89^ This disparity can be illustrated by the real‐world use of CAR T cell therapy for diffuse large B‐cell lymphoma (DLBCL) in the US healthcare system, which leads the world in CAR T cell therapy development and implementation. A 2021 report from the Center for International Blood and Marrow Transplant Research, which accounts for approximately 95% of all T cell therapies administered at US centers, reported only 2000 CAR T cell treatments performed that year. In contrast, it is estimated that over 10,000 patients in the US develop relapsed or refractory DLBCL annually and could potentially benefit from such therapies.^99^ This gap between the number of eligible patients and those who receive treatment reflects significant access limitations, even in high‐resource settings. The disparity is even more pronounced globally, where access to CAR T cell therapies remains extremely limited. Additionally, even among patients who gain access to CAR T cell therapy, a significant number may drop out mid‐process due to disease progression during the waiting period, a consequence of the lengthy and complex manufacturing timeline. For example, in a clinical trial (NCT03435796) involving autologous T cell therapy, 9% of patients who underwent leukapheresis for T cell collection ultimately did not proceed to infusion, often due to clinical deterioration during the manufacturing window.^217^ Beyond logistical challenges, the high financial burden of therapy significantly limits access to T cell treatments for a broader patient population. In the United States, a single infusion of CAR T cell therapy is priced between $373,000 and $475,000, making it inaccessible for many patients even in high‐income settings.^218^ In response to these barriers, developing countries such as India have taken steps to develop homegrown CAR T cell products at a fraction of the cost, reportedly as low as one‐tenth of the United States price.^89^ These efforts are positioning India as a leader in the development and implementation of low‐cost T‐cell therapies, potentially paving the way for more affordable global access.^98^, ^99^

To address the logistical challenges associated with the implementation of T cell therapies, several innovative approaches are being explored to improve accessibility and streamline clinical workflows, particularly through the development of decentralized therapeutic protocols.^219^ One promising strategy is the use of on‐site, fully automated manufacturing platforms that enable the production of fresh T cell products.^89^ This approach eliminates the need for cryopreservation and cold chain logistics, thereby reducing both cost and complexity. Moreover, fresh products are expected to offer higher cell viability, leading to more rapid in vivo expansion and enhanced therapeutic efficacy compared to frozen counterparts. A clinical trial (NCT01822652) is currently underway to directly compare fresh versus cryopreserved T cell products, aiming to evaluate the impact of this manufacturing improvement on clinical outcomes, cell fitness, and overall treatment feasibility. In addition, emerging manufacturing technologies are poised to significantly reduce T cell production timelines, cutting the manufacturing process from weeks to just a few days and thereby accelerating patient access to therapy. One example is the T‐Charge™ platform, which enables the generation of anti‐CD19 CAR T cell product YTB323 with less than 2 days of ex vivo culture, compared to the traditional 1–2 weeks required for conventional CAR T cell products.^219^, ^220^ This accelerated manufacturing approach is currently being evaluated in multiple clinical trials, including YTB323 (NCT06617793, NCT05798117, and NCT06704269) and FasT CAR T cells (NCT06327997, NCT06249256, and NCT05886738). These innovations aim to limit costs, reduce manufacturing failures, and expand access by enabling faster turnaround times for eligible patients. Recent advancements have also focused on shifting the paradigm from ex vivo to in vivo generation of T cell therapies, aiming to simplify manufacturing and broaden access. This transformative approach involves delivering genetic material directly into the body to engineer antigen‐specific T cells in situ, eliminating the need for complex ex vivo manipulation. Several delivery platforms have been explored in preclinical studies, including viral vectors such as lentivirus and adeno‐associated virus, as well as non‐viral carriers like lipid nanoparticles and polymeric nanocarriers. These platforms have demonstrated the ability to generate functional, antigen‐specific T cells in vivo.^161^ Some of these technologies have now progressed into early‐phase clinical trials, including lentiviral vectors delivering BCMA CAR constructs (NCT06791681, NCT06691685) and lentiviral vectors delivering CD19 CAR constructs (NCT06528301). These studies represent a new horizon toward streamlined, off‐the‐shelf T cell therapies and may hold the key to enabling wider global adoption.^219^

## 6 CONCLUSION

With their targeted effector functionality and long‐term persistence, T cell therapies offer unique therapeutic advantages and have emerged as the new frontier in cellular medicine. They have surpassed stem cell therapies in both the number of approved products over the past 5 years and currently active clinical trials, marking a pivotal shift in the landscape of cell therapies. Since their initial characterization in the 1960s, there has been remarkable growth in our understanding of T cell biology, engineering, and pathophysiological relevance, insights that have directly motivated their evolution into powerful clinical entities. To date, many T cell therapies have received regulatory approval across multiple regions, including the United States, the European Union, India, China, and others. Our analysis identified 19 distinct approved T cell therapy products, encompassing a range of T cell types such as TILs, TCR‐transduced, CAR‐transduced, virus‐specific T cells, and LAK cells. These therapies address a diverse array of indications, including hematologic malignancies, solid tumors, and transplant‐related disorders. The collective features of these approved products reflect the key principles supporting the clinical success of T cell therapies, which in turn have been shaping the past, current, and future clinical landscape of T cell therapies. Through the analysis of 2570 clinical trials involving T cell‐based interventions, we identified three distinct phases of clinical and scientific progression in T cell therapy development including 1990–2000: foundational studies and early priming/expansion strategies, 2001–2012: the emergence of genetic engineering and lymphodepleting conditioning, marking the first major inflection point; 2013–2025: rapid clinical expansion, driven by the clinical success of CD19 CAR T cells, marking the second major inflection point. Over these stages of development, the clinical trial landscape for T cell therapies has evolved significantly across multiple dimensions, including trial sponsorship, T cell type and source, disease indication, delivery route, ex vivo modification strategies, targeting approaches, and conditioning regimens. Collectively, these changes reflect the expanding scope and growing sophistication of T cell therapies as they progress toward broader clinical translation. Despite these substantial advancements, the wider adoption of T cell therapies across new disease indications and diverse patient populations remains constrained by persistent challenges. These include scientific hurdles such as functional decline, impaired trafficking, and target antigen heterogeneity post‐adoptive transfer, as well as technological limitations involving cell sourcing and the complexity of manufacturing protocols. These challenges are now at the forefront of ongoing research, driving innovation aimed at fully realizing the therapeutic potential of T cell therapies. Emerging strategies such as in vivo engineering, mRNA‐based editing, data‐driven logic‐gated gene constructs, and off‐the‐shelf cell banking approaches represent key advancements that may drive the next major inflection point in the evolution of T cell therapies. With their proven clinical efficacy, continued progress in clinical development, and a growing array of technological innovations, T cell therapies remain a dynamic and rapidly advancing field. Their application is expected to expand even further in the years ahead, offering transformative possibilities for patients across a wide variety of diseases and diverse geographical settings.

## AUTHOR CONTRIBUTIONS

**Suyog Shaha:** Conceptualization; methodology; investigation; data curation; formal analysis; visualization; writing—original draft; writing review and editing. **Leah Lourenco:** Investigation; data curation; writing review and editing. **Zongmin Zhao**: Conceptualization; funding acquisition; writing review and editing. **Samir Mitragotri:** Conceptualization; funding acquisition; writing review and editing.

## CONFLICT OF INTEREST STATEMENT

SM and ZZ are inventors on patent applications in the field of cell therapies (owned and managed by Harvard University).

## Data Availability

The data that support the findings of this study are available from the corresponding author upon reasonable request.
